# Development and Validation of a Mixture-of-Experts–Based Model for Postoperative In-Hospital Mortality Risk Assessment: Multicenter Retrospective Study

**DOI:** 10.2196/92856

**Published:** 2026-11-04

**Authors:** Shixin Huang, Jinhong Xia, Xixi Nie, Zhuo Zheng, Jiawei Luo

**Affiliations:** 1 Department of Scientific Research, The People's Hospital of Yubei District of Chongqing city, Chongqing, China; 2 Network Technology and Education Digitalization Center, Sichuan College of Traditional Chinese Medicine, Mianyang, China; 3 School of Artificial Intelligence, Chongqing University of Posts and Telecommunications, Chongqing, China; 4 Department of Anesthesiology, Chengdu Second People's Hospital, Chengdu, Sichuan, China; 5 West China Biomedical Big Data Center, West China Hospital of Sichuan University, No. 37, Guoxue Alley, Wuhou District, Chengdu, Sichuan, 610041, China, 86 18782146800

**Keywords:** postoperative in-hospital mortality, perioperative risk prediction, surgical outcomes, mixture of experts, intraoperative time-series data

## Abstract

**Background:**

Postoperative mortality remains a key indicator of surgical safety and quality. Traditional risk models based on static preoperative or intraoperative variables often fail to capture dynamic physiological changes during surgery and generalize poorly across surgical types. To address these limitations, we developed and validated a deep learning framework capable of learning heterogeneous temporal patterns from intraoperative signals while maintaining interpretability and adaptability.

**Objective:**

This study aimed to develop a mixture-of-experts–based model (Postoperative Risk Modeling Expert network, PRIME-MoE) to provide dynamic, surgery-adaptive postoperative in-hospital mortality risk prediction. The primary objective was to improve prediction accuracy, cross-database robustness, and interpretability across heterogeneous surgical populations and institutions.

**Methods:**

We retrospectively analyzed 173,990 surgical cases from the INSPIRE (Informative Surgical Patient Dataset for Innovative Research Environment) and HUAXI databases. PRIME-MoE used 4 one-layer unidirectional long short-term memory experts with dense softmax routing based on static surgical context. We evaluated discrimination, calibration, threshold-specific operating points, parameter count, graphics processing unit inference latency, routing patterns, and a sensitivity analysis that truncated the final 30 minutes of each operation without retraining.

**Results:**

PRIME-MoE achieved internal-test area under the receiver operating characteristic curves (AUROCs) of 93.8 (95% CI 92.3‐96.0) in INSPIRE and 90.7 (95% CI 87.1‐94.8) in HUAXI. Reciprocal evaluation retained discrimination (AUROCs 90.8 and 87.8) but showed imperfect calibration (Brier scores 0.035‐0.049; calibration slopes 0.28‐0.49), supporting site-specific recalibration. Graphics processing unit inference required a median of 11.4 ms (9.1-18.0; 3.3-45.3) per case. Without retraining, scoring 30 minutes before the recorded end of surgery retained AUROCs of 93.1 in INSPIRE and 90.2 in HUAXI.

**Conclusions:**

PRIME-MoE provides an interpretable framework for postoperative in-hospital mortality prediction and retained discrimination in reciprocal evaluation across 2 heterogeneous perioperative databases. The 30-minute analysis supports near-end-of-surgery scoring, but calibration was insufficient for direct deployment. Prospective validation, local recalibration, threshold selection, and workflow evaluation are required before clinical use.

## Introduction

Postoperative mortality is one of the most critical clinical indicators in modern surgery and anesthesiology [1,2]. Surgical procedures impose intense physiological stress on the body and trigger complex systemic responses within a short period. Early identification of patients at high risk of postoperative death is essential for targeted monitoring, timely intervention, and rational allocation of intensive care resources. Accurate prediction of postoperative in-hospital mortality can help clinicians optimize postoperative management, guide resource allocation, and improve overall surgical outcomes. During surgery, monitoring systems continuously record large volumes of high-frequency physiological signals such as blood pressure, heart rate, oxygen saturation, respiratory parameters, and drug infusion rates. These time-series data reflect the dynamic physiological status of the patient, yet their complexity and volume make it difficult for clinicians to capture all critical changes in real time [3-6]. Important fluctuations or latent risk patterns may be missed. AI methods, especially those designed for time-series analysis, can automatically extract and interpret these dynamic patterns, offering a more comprehensive and objective basis for postoperative in-hospital mortality prediction [1,7-12].

Previous studies have developed perioperative risk prediction models using preoperative variables, intraoperative summaries, or routinely available electronic health record (EHR) data, including multicenter machine learning models [1,10,13-17]. These models often rely on static features extracted from preoperative and intraoperative data, such as mean or extreme values of vital signs, durations below specific thresholds (eg, hypotension), and total drug or fluid dosages. However, compressing rich intraoperative trajectories into static summaries inevitably loses crucial temporal information, making it difficult to capture when instability occurs, how long it lasts, or how interventions dynamically affect hemodynamics, which are factors essential for understanding postoperative outcomes. Recent advances in time-series modeling, including long short-term memory (LSTM), gated recurrent units (GRUs), and transformer networks, enable learning of complex temporal dependencies from high-frequency physiological signals and offer a more dynamic representation of patient status [18-21]. However, a single global temporal model may still have difficulty capturing heterogeneous intraoperative patterns across diverse surgical procedures and institutional settings.

Despite prior progress, surgical heterogeneity remains an important challenge for model generalization and clinical interpretation [11,12,22-24]. Different procedures, such as cardiac, neurosurgical, and general surgeries, exhibit distinct physiological patterns, intervention strategies, and risk thresholds [25-27]. The same intraoperative variable (eg, mean arterial pressure or end-tidal CO_₂_) may have different clinical meanings across surgical types. As a result, a single unified model may not fully adapt to surgery-adaptive dynamics across diverse surgical populations and clinical centers. Training separate models for each surgery may better capture local characteristics but can lead to smaller sample sizes and reduced statistical stability. Therefore, a modeling strategy that balances shared perioperative information with surgery-adaptive specialization may be useful for heterogeneous perioperative prediction tasks.

Mixture-of-experts (MoE) architectures have increasingly been explored for modeling heterogeneity in longitudinal clinical data. Lee and Hauskrecht [28] developed a residual MoE framework in which multiple GRU-based experts captured patient-subpopulation–specific deviations from a population-level model for clinical event sequence prediction. More recently, MIRA incorporated a frequency-specific sparse MoE layer to route irregular medical time-series representations across experts specializing in different temporal regimes [29]. The MANDARIN framework integrated static and temporal intensive care unit (ICU) data using an MoE architecture for dynamic prediction of delirium and coma across multiple clinical cohorts [30]. Collectively, these studies demonstrate that expert routing can help represent heterogeneous temporal patterns in clinical data. However, they focused primarily on general EHR event prediction, medical signal forecasting, or ICU neurological state prediction rather than postoperative mortality prediction from complete intraoperative multivariate trajectories. Beyond clinical prediction, MoE research has addressed heterogeneous sequential prediction, expert routing and specialization, scalable model design, and expert attribution across other domains [31-35]. These broader studies inform architectural design but do not establish clinical use.

To extend this line of research, we developed Postoperative Risk Modeling Expert network (PRIME-MoE), a perioperative MoE model that combines multivariate intraoperative time-series data with static patient and surgical context. Four 1-layer unidirectional LSTM experts process the dynamic sequence, while a dense softmax router derived from the static context forms a weighted expert representation for postoperative in-hospital mortality prediction. We evaluated internal and reciprocal external discrimination, calibration, threshold-specific operating characteristics, computational cost, routing behavior, interpretability, and the feasibility of scoring 30 minutes before the recorded end of surgery.

## Methods

The completed TRIPOD+AI (Transparent Reporting of a multivariable prediction model for Individual Prognosis Or Diagnosis + Artificial Intelligence) reporting checklist is provided in Checklist 1.

### Ethical Considerations

The INSPIRE (Informative Surgical Patient Dataset for Innovative Research Environment) database is a publicly available perioperative dataset hosted on PhysioNet and developed by Seoul National University Hospital (SNUH). It contains deidentified surgical data collected from 2011 to 2020 with prior institutional review board approval. The HUAXI database was retrospectively constructed from electronic medical records at West China Hospital, Sichuan University, covering surgical patients from 2018 to 2024. As this study involved secondary analysis of deidentified data, the requirement for informed consent was waived by the Institutional Review Board of West China Hospital, Sichuan University (approval number 20190473). Both databases were used in accordance with the principles of the Declaration of Helsinki and relevant ethical and data protection standards.

### Data Sources

We used 2 perioperative databases in this study: INSPIRE [36], a publicly available dataset for perioperative medicine research, and a retrospective database from West China Hospital of Sichuan University, referred to as the HUAXI database. Both databases contain detailed surgical information, diagnostic data, perioperative vital signs, anesthesia monitoring data, laboratory test results, and medication records. The INSPIRE dataset was collected at Seoul National University Hospital in South Korea between 2011 and 2020 and includes approximately 130,000 surgeries from about 99,000 patients. From this dataset, we extracted 99,735 surgical cases, each uniquely identified by an operation ID (“op_id”). The HUAXI database includes surgical patients treated at West China Hospital between 2018 and 2024, all of whom had complete in-hospital follow-up until discharge. We extracted 83,428 surgical cases, each also uniquely identified by an “op_id.” In both datasets, one surgery was treated as the analysis unit. We excluded cases involving patients younger than 18 years, surgeries with invalid start or end times, nongeneral anesthesia procedures, and surgeries lasting less than 15 minutes. After filtering, 99,735 surgeries from the INSPIRE dataset and 74,255 surgeries from the HUAXI database were included in the final analysis.

### Outcome Definition

The primary outcome of this study was postoperative in-hospital mortality. For each eligible surgical case, postoperative in-hospital mortality was defined as death occurring after the end of surgery and before discharge from the index hospitalization. The same outcome definition was applied to both the INSPIRE and HUAXI databases to ensure consistency in model development, internal testing, and cross-database validation. In the present study, we did not use 30-day or 90-day mortality because these longer-term follow-up outcomes were not consistently available across the 2 databases.

### Variable Preprocessing

We aligned the measurement units of laboratory tests between the 2 databases and standardized the coding and units of intraoperative medication variables. The corresponding alignment scripts are available in the code repository associated with this study. The input data included patient demographics, preoperative comorbidities, the most recent laboratory measurements before surgery, intraoperative physiological variables, and intraoperative medications and fluids, as summarized in Table S7 in Multimedia Appendix 1. Surgical procedures were harmonized into 9 broad anatomical categories to construct a consistent surgical-site variable across the 2 databases. The Other category was used as a residual harmonization category for procedures that could not be assigned to the 8 principal anatomical categories. In both INSPIRE and HUAXI, this category included procedures involving the Peripheral Vascular System, Lymphatic and Hemic Systems, source-labeled Other procedures, and other procedures that could not be consistently mapped to one of the principal anatomical groups. The same harmonization principle was applied to both databases. Therefore, the Other category should be interpreted as a heterogeneous residual grouping rather than a clinically homogeneous procedural category. Because intraoperative physiological and treatment data were recorded longitudinally at irregular intervals, a standardized preprocessing procedure was required before temporal modeling. To reduce the influence of data-entry and recording errors, continuous values outside predefined clinically plausible ranges were treated as missing. Invalid categories and inconsistent data types for categorical variables were also recoded as missing before subsequent processing.

We processed irregular longitudinal data using the previously published Electronic Medical Record-Longitudinal Irregular Preprocessing (EMR-LIP) framework proposed by Luo et al [37]. EMR-LIP applies a variable-type-specific and observation-aware procedure to transform irregular electronic medical record data into regularly aligned sequences suitable for deep learning. The duration of each operation was divided into consecutive 5-minute intervals from the recorded start to the end of surgery. For each dynamic variable within each interval, directly recorded source measurements were retained. When multiple numerical observations were available within the same interval, their median was used; when multiple categorical observations were available, their mode was used. If no source measurement was recorded for a variable within a given interval, the corresponding value was initially marked as missing.

Missing intervals were subsequently processed according to the variable type and the temporal position of the missing value rather than by applying a single imputation method to all variables. For numerical variables, internal gaps between 2 observed measurements were completed using linear interpolation. Leading missing intervals before the first available measurement were completed using the first available intraoperative observation for that variable, whereas trailing missing intervals after the last available measurement were completed by carrying the last observed value forward. When a variable had no valid observation throughout an operation, the training-set median was used for numerical variables and the training-set mode was used for categorical variables. Intermittently missing categorical values were represented using a distinct missing category. Time points at which no dynamic variable had any source observation were removed from the sequence.

In addition to the completed value sequence, a binary observation mask was generated for every variable and time point to preserve information on whether a value was directly observed or completed during preprocessing. For variable *x*_*d*_ at time point *t_i_*, the mask was defined as $m_{t_{i},d=0}$ when at least one original source measurement was available within the corresponding interval and $m_{t_{i},d=1}$ otherwise. This mask therefore retained the original observation pattern separately from the completed values.

The percentages reported in Table S8 in Multimedia Appendix 1 describe interval-level recording density after construction of the regular 5-minute time grid. Specifically, an observed interval contained at least one original source measurement for the corresponding variable, whereas a completed interval contained no newly recorded source measurement and was processed using the variable-specific EMR-LIP procedure described above. These percentages should not be interpreted as patient-level missingness rates or as indicating that an entire patient trajectory was synthetically generated. In irregular perioperative data, a variable may be available and clinically monitored during an operation without a new value being entered in every 5-minute interval.

Missing values in static variables, including demographics, medical history, and the most recent preoperative laboratory results, were imputed using predictive mean matching. All imputation parameters and training-set summary statistics were estimated using the training data only and were subsequently applied to the validation and test sets to avoid information leakage.

For conventional machine learning models that could not directly process longitudinal sequences, each intraoperative continuous variable was summarized using 8 statistical features: minimum, maximum, median, mean, SD, IQR, skewness, and kurtosis.

### Model Development

We developed the PRIME-MoE to estimate postoperative in-hospital mortality from preoperative context and intraoperative time-series data. We use the term surgery-adaptive to mean that static surgical context, including surgical category and American Society of Anesthesiologists (ASA) class, determines patient-specific routing weights over temporal experts that process the complete intraoperative sequence; the model does not train a separate predictor for each procedure group.

Each expert module was implemented as a 1-layer unidirectional LSTM encoder with hidden size 128. The dynamic input contained 143 features per 5-minute interval, including observation masks. Static preoperative features (111 dimensions, including demographics, ASA indicators, surgical-category indicators, preoperative laboratory values, and missingness indicators) were processed by a 2-layer multilayer perceptron (MLP) with hidden size 64. A separate router MLP with hidden size 32 mapped the same static vector to *K* nonnegative weights using softmax. The weighted expert embedding was concatenated with the static embedding and passed through a prediction MLP with hidden size 128 and a sigmoid output. The final models used *K*=4 experts, soft dense routing in which all experts contributed according to their router weights, and no dropout layers.

Models were optimized with Adam (learning rate 0.001, weight decay 0.001) using weighted binary cross-entropy to address class imbalance. The batch size was 150. Patient-stratified training, validation, and test splits were created in a 7:2:1 ratio, and early stopping was based on validation area under the receiver operating characteristic curve (AUROC). Sensitivity analyses for *K* in {1, 2, 3, 4, 5} are reported in Multimedia Appendix 1.

### Operational Feasibility and Earlier Prediction Horizons

The present study is retrospective. The primary prediction time point was the recorded end-of-surgery timestamp after offline EMR-LIP preprocessing. This design evaluates the information available in complete intraoperative trajectories but does not measure prospective data availability, streaming latency, or workflow integration.

For prospective deployment, the intended use is early postoperative risk stratification near the anticipated end of surgery, such as during wound closure, when postanesthesia care unit (PACU) or higher-acuity disposition decisions are often finalized. Minimum inputs would include the preoperative static features used by the model and intraoperative variables aligned to the 5-minute EMR-LIP grid from surgery start to the selected trigger time.

To test an earlier trigger without retraining, we removed the final six 5-minute bins from each intraoperative sequence, retained at least 1 bin for short operations, and generated scores with the end-of-surgery-trained weights. This analysis therefore represents scoring 30 minutes before the recorded end of surgery rather than a separately trained 30-minute model.

Neural-network inference latency was benchmarked on an NVIDIA L20 graphics processing unit (GPU). On 300 real INSPIRE test sequences, median per-case scoring time for PRIME-MoE (*K*=4) was 11.4 ms (9.1-18.0; 3.3-45.3)(mean 13.1; SD 7.0; P95 25.8 ms), excluding EHR extraction and EMR-LIP preprocessing.

### Model Interpretation

To examine how inputs contributed to PRIME-MoE predictions, we applied integrated gradients (IG) [38]. IG attributes the difference between a model output and a defined baseline to individual input features along a gradient path. The method is computationally tractable for the high-dimensional LSTM-based sequence model and satisfies the completeness property. These attributions describe model dependence and were not interpreted as causal effects.

Formally, for a model *F* and an input vector *x*, IG measures the accumulated gradients of *F* with respect to *x* along a straight-line path from a baseline input *x*′ (often a zero tensor) to *x*. This ensures that feature attributions are consistent and satisfy key properties such as completeness and sensitivity, making IG a robust and theoretically sound choice for medical deep learning interpretation.

The IG for input *x* is defined as follows:


$$IntegratedGrads(x)=(x-x^{\prime })\times \int_{\alpha =0}^{1}\frac{\partial F\left(x^{\prime }+\alpha (x-x^{\prime })\right)}{\partial x}\;d\alpha$$

where *x*^′^ represents the baseline input and *α* defines the interpolation path between *x*′ and *x*. In practice, this integral is numerically approximated as follows:


$$IntegratedGrads(x)\approx (x-x^{\prime })\times \frac{1}{m}\sum_{k=1}^{m}\frac{\partial F\left(x^{\prime }+\frac{k}{m}(x-x^{\prime })\right)}{\partial x}$$

where *m* is the number of integration steps used for the approximation. In our implementation, the baseline *x′* was set to a zero tensor in the standardized model-input space. Because continuous variables were standardized using *z*-score normalization based on the mean and SD calculated from the training set, a value of zero in the transformed space corresponds to the training-set mean of that variable in the original scale. Therefore, the IG baseline represented an average reference state derived from the training data rather than physiologically impossible values such as a heart rate or blood pressure of zero.

By applying IG, we were able to visualize and quantify the attribution of preoperative features and intraoperative time-series signals to the predicted risk of postoperative in-hospital mortality. This approach provided transparent explanations for the model’s decision-making process and helped identify clinical features associated with model predictions, while not implying causal effects of individual variables on mortality.

### Model Evaluation

We evaluated discrimination using AUROC and area under the precision-recall curve (AUPRC) and reported accuracy, recall, precision, and *F*_1_-score at prespecified operating points. Calibration was quantified using the Brier score, logistic calibration slope and intercept (outcome regressed on the logit of predicted probability), and 10-bin expected calibration error. Reliability diagrams were generated for internal and reciprocal evaluations. Subgroup analyses examined broad surgical categories and ASA physical status groups.

For each subgroup, performance was evaluated independently using the same set of metrics. The 95% CIs were estimated through bootstrapping with 1000 resamples. The evaluation metrics were defined as follows:


$$\text{Accuracy}\text{=}\frac{\text{TP+TN}}{\text{TP+TN+FP+FN}}$$

$$\text{Recall}\text{=}\frac{\text{TP}}{\text{TP+FN}}$$

$$\text{Precision}\text{=}\frac{\text{TP}}{\text{TP+FP}}$$

$$\text{F}\text{1 }\text{Score}\text{=}\frac{\text{Precision}\text{×}\text{Recall}}{\text{Precision}\text{+}\text{Recall}}$$

where TP denotes true positives, TN true negatives, FP false positives, and FN false negatives. By evaluating PRIME-MoE across surgical and ASA-based subgroups, we aimed to verify its generalizability and fairness in capturing in-hospital mortality risk across heterogeneous patient populations and surgical types. Pairwise comparisons of AUROC between PRIME-MoE and LSTM within each subgroup were performed using DeLong’s test. Because these subgroup analyses were exploratory, unadjusted *P* values are reported. Interpretation focused primarily on point estimates, 95% CIs, subgroup sample sizes, and outcome event counts rather than on statistical significance. No adjustment for multiple comparisons was applied.

### Threshold-Oriented Evaluation

Because postoperative in-hospital mortality is rare, we did not recommend a universal threshold. We evaluated Youden and *F*_1_-maximizing thresholds and operating points targeting sensitivities of at least 80%, 90%, and 95%. For clinical use, we propose a site-specific process: estimate local prevalence and acceptable alerts per 1000 procedures; select a sensitivity target appropriate to the use case; determine and freeze the corresponding threshold on a local validation set; recalibrate probabilities when slope or intercept indicates miscalibration; and monitor positive predictive value, false positives per 1000 procedures, and missed events after deployment. *F*_1_-maximizing thresholds are reported for completeness but are not proposed as default clinical cutoffs.

## Results

### Baseline Characteristics of the Cohort

To build the model, we included a total of 173,990 inpatient records of surgeries performed under general anesthesia from 2 databases, with 99,735 cases from the INSPIRE database and 74,255 cases from the HUAXI database (Figure 1). Table 1 presents the baseline characteristics of the included patients from both databases, using each surgery record as the unit of analysis. Table S1 in Multimedia Appendix 1 shows the baseline characteristics of the training, validation, and test sets from INSPIRE and HUAXI. Among all included cases, the overall postoperative in-hospital mortality rate was 0.50% (862/173,990), including 0.58% (577/99,735) in INSPIRE and 0.38% (285/74,255) in HUAXI.

**Figure 1.**
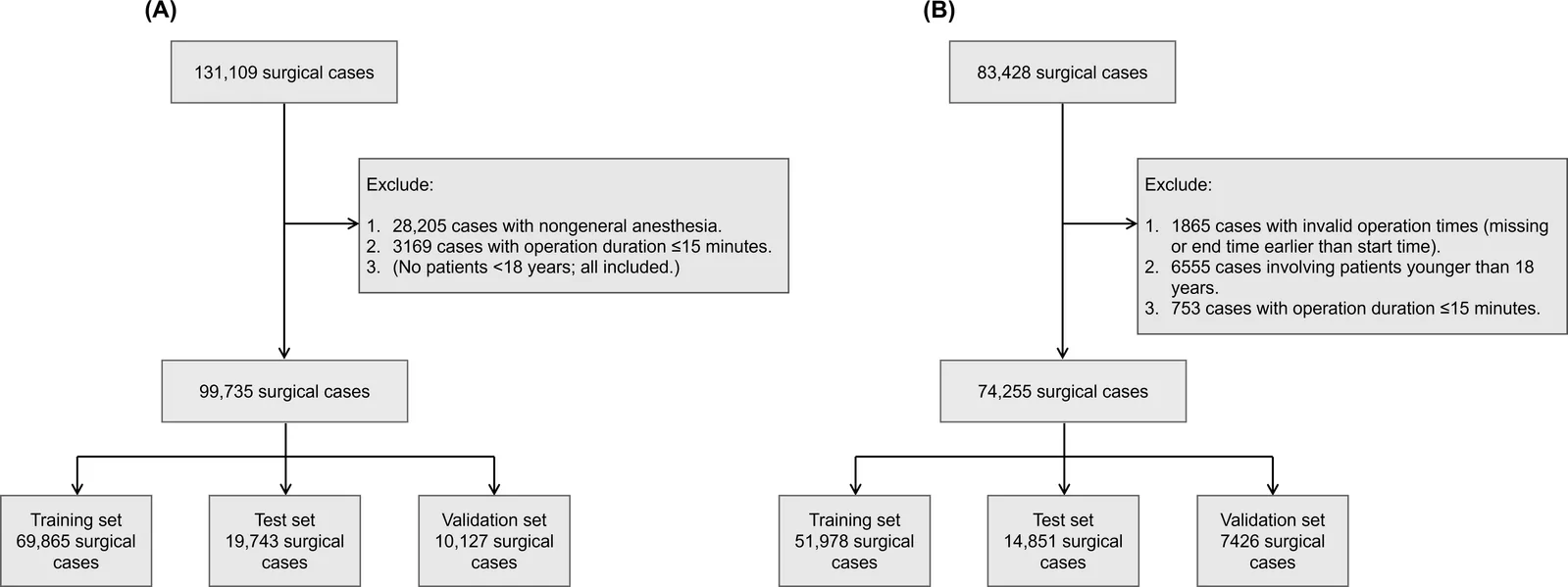
(A) Sample selection process in the INSPIRE (Informative Surgical Patient Dataset for Innovative Research Environment) database. (B) Sample selection process in the HUAXI database.

**Table 1.** Demographic characteristics of selected samples from different databases.

Characteristics	Overall	INSPIRE^a^	HUAXI
Operations, n	173,990	99,735	74,255
Age (y), mean (SD)	56.99 (15.69)	54.61 (15.68)	60.19 (15.13)
Sex, n (%)			
Female	88,260 (50.73)	54,857 (55)	33,403 (44.98)
Male	85,729 (49.27)	44,878 (45)	40,851 (55.02)
BMI (kg/m^2^), mean (SD)	23.54 (3.47)	23.65 (3.47)	23.39 (3.48)
ASA^b^ grade, n (%)			
1‐2	135,378 (79.05)	87,045 (89.74)	48,333 (65.09)
3‐4	35,739 (20.87)	9851 (10.16)	25,888 (34.86)
≥5	140 (0.08)	106 (0.11)	34 (0.05)
Emergency, n (%)			
No	160,001 (91.96)	89,761 (90)	70,240 (94.59)
Yes	13,989 (8.04)	9974 (10)	4015 (5.41)
Surgery sites, n (%)			
Abdomen	59,669 (34.29)	21,837 (21.9)	37,832 (50.95)
Cardiovascular	4819 (2.77)	2777 (2.78)	2042 (2.75)
Central nervous system	5361 (3.08)	4681 (4.69)	680 (0.92)
Head and neck	19,541 (11.23)	16,281 (16.32)	3260 (4.39)
Musculoskeletal	20,912 (12.02)	9533 (9.56)	11,379 (15.32)
Other	12,708 (7.3)	11,725 (11.76)	983 (1.32)
Respiratory	13,656 (7.85)	4509 (4.52)	9147 (12.32)
Skin and soft tissue	15,934 (9.16)	13,166 (13.2)	2768 (3.73)
Urinary and reproductive	21,390 (12.29)	15,226 (15.27)	6164 (8.3)
Duration of surgery (min), mean (SD)	136.55 (104.35)	136.44 (110.35)	136.71 (95.70)
Duration of anesthesia (min), mean (SD)	186.55 (118.52)	174.05 (120.93)	203.33 (113.05)
Postoperative in-hospital death, n (%)			
Survived	173,128 (99.5)	99,158 (99.42)	73,970 (99.62)
Died	862 (0.5)	577 (0.58)	285 (0.38)

a INSPIRE: Informative Surgical Patient Dataset for Innovative Research Environment.

b ASA: American Society of Anesthesiologists.

### Model Performance on the INSPIRE and HUAXI Databases

After conducting the sensitivity analysis of *K*, we found that the overall performance of the model no longer improved when *K* exceeded 4. Therefore, we set *K* to 4 for the subsequent analyses (Figure S1 in Multimedia Appendix 1). In the postoperative in-hospital mortality prediction task triggered at the end of surgery, the PRIME-MoE model consistently outperformed all baseline models, including ASA, logistic regression, extreme gradient boosting (XGBoost), and LSTM (Table 2). On the INSPIRE database, PRIME-MoE achieved an AUROC of 93.8 (95% CI 92.3‐96.0) and an AUPRC of 22.2 (95% CI 16.6‐29.8), outperforming the LSTM model, which achieved an AUROC of 91.1 (95% CI 88.8‐92.9) and an AUPRC of 17.8 (95% CI 13.6‐24.5). Similarly, on the HUAXI database, PRIME-MoE achieved an AUROC of 90.7 (95% CI 87.1‐94.8) and an AUPRC of 6.4 (95% CI 4.4‐10.4), compared with an AUROC of 88.5 (95% CI 85.5‐91.8) and an AUPRC of 4.3 (95% CI 2.4‐6.9) for the LSTM baseline. These results suggest that the MoE framework improved overall discrimination and precision-recall performance across both datasets. However, given the very low incidence of postoperative in-hospital mortality, especially in the HUAXI cohort, threshold-dependent metrics such as recall, precision, and *F*_1_-score should be interpreted with caution.

**Table 2.** Summary of model performance across the two databases.

Database and model	AUROC^a^ (95% CI)	AUPRC^b^ (95% CI)	Accuracy (95% CI)	Recall (95% CI)	Precision (95% CI)	*F*_1_-score (95% CI)
INSPIRE^c^						
ASA^d^	84.7 (80.8-87.8)	22.4 (16.4-30.8)	99.5 (99.4-99.6)	16.4 (0.0-24.2)	94.7 (0.0-100.0)	27.9 (0.0-38.6)
LR^e^	86.9 (84.0-88.8)	16.2 (12.2-22.8)	99.1 (98.9-99.3)	30.5 (24.9-41.9)	31.4 (21.9-39.8)	30.9 (25.0-38.7)
XGBoost^f^	88.9 (85.8-91.2)	15.9 (10.1-23.2)	99.1 (98.4-99.3)	28.9 (24.0-43.9)	30.8 (18.8-41.1)	29.8 (23.7-37.4)
LSTM^g^	91.1 (88.8-92.9)	17.8 (13.6-24.5)	99.2 (98.4-99.3)	28.1 (23.6-53.5)	34.0 (18.8-42.2)	30.8 (24.4-36.3)
PRIME-MoE^h^	93.8 (92.3-96.0)	22.2 (16.6-29.8)	98.8 (98.7-99.3)	43.8 (29.1-52.5)	25.8 (21.6-40.1)	32.5 (26.5-38.8)
HUAXI						
ASA	81.5 (77.2-86.7)	5.7 (2.2-13.0)	99.6 (99.6-99.7)	3.8 (0.0-8.8)	50.0 (0.0-100.0)	7.0 (0.0-15.3)
LR	85.9 (81.3-89.4)	2.7 (1.6-5.1)	97.9 (97.1-99.1)	26.4 (4.5-34.1)	5.0 (2.1-8.1)	8.3 (2.9-12.2)
XGBoost	86.8 (84.1-90.5)	3.1 (1.9-6.2)	98.2 (97.8-99.3)	26.4 (7.4-38.0)	5.8 (3.0-9.6)	9.5 (5.2-14.0)
LSTM	88.5 (85.5-91.8)	4.3 (2.4-6.9)	98.7 (97.3-99.3)	24.5 (9.5-39.3)	7.7 (3.5-11.2)	11.7 (6.2-15.5)
PRIME-MoE	90.7 (87.1-94.8)	6.4 (4.4-10.4)	99.3 (97.9-99.4)	17.0 (11.5-45.7)	13.4 (6.9-20.1)	15.0 (9.1-20.7)

a AUROC: area under the receiver operating characteristic curve.

b AUPRC: area under the precision-recall curve.

c INSPIRE: Informative Surgical Patient Dataset for Innovative Research Environment.

d ASA: American Society of Anesthesiologists.

e LR: logistic regression.

f XGBoost: extreme gradient boosting.

g LSTM: long short-term memory.

h PRIME-MoE: Postoperative Risk Modeling Expert network.

Threshold-dependent metrics were calculated using model-specific thresholds selected by maximizing the *F*_1_-score in the validation set rather than using a universal threshold of 0.5.

### Operational Feasibility and Earlier Prediction

Using the end-of-surgery-trained models without retraining, removal of the final 30 minutes of intraoperative data yielded AUROCs of 0.931 (AUPRC 0.205; Brier score 0.048) in INSPIRE and 0.902 (AUPRC 0.065; Brier score 0.038) in HUAXI, compared with end-of-surgery AUROCs of 0.937 and 0.907, respectively. The absolute AUROC reductions were 0.006 and 0.005. A full 30-minute lead time was unavailable for 4.9% of INSPIRE and 7.7% of HUAXI test cases; these short operations retained at least one time bin (Table S14 in Multimedia Appendix 1).

On 300 real INSPIRE test cases, PRIME-MoE required a median of 11.4 ms (9.1-18.0; 3.3-45.3) per case on GPU (mean 13.1, SD 7.0; P95 25.8 ms; P99 33.8 ms). These measurements exclude EHR extraction and preprocessing, which remain the principal unmeasured contributors to end-to-end clinical latency (Table S12 in Multimedia Appendix 1).

### Threshold-Oriented Performance and False-Positive Burden

Because postoperative in-hospital mortality was rare, we further evaluated PRIME-MoE and LSTM at matched high-sensitivity operating points rather than relying on a single fixed threshold (Table 3). In the HUAXI test set, at a sensitivity of 81.1%, PRIME-MoE achieved a specificity of 87.2% and a PPV of 2.2%, compared with 79.0% and 1.4%, respectively, for LSTM. PRIME-MoE generated 127.2 false-positive predictions per 1000 procedures, compared with 209.1 for LSTM, corresponding to 81.9 fewer false positives per 1000 procedures. Both models missed 10 deaths at this operating point.

**Table 3.** Threshold-oriented comparison of PRIME-MoE^a^ and long short-term memory (LSTM) at matched high-sensitivity operating points^b^.

Database and target sensitivity	Model	Threshold	Sensitivity	Specificity	PPV^c^	FP^d^ per 1000	FN^e^
HUAXI							
80	LSTM	0.0891	81.1	79	1.4	209.1	10
80	PRIME-MoE	0.025	81.1	87.2	2.2	127.2	10
90	LSTM	0.0226	90.6	69.1	1	308.4	5
90	PRIME-MoE	0.0036	90.6	76.4	1.4	235.1	5
INSPIRE^f^							
80	LSTM	0.1024	80.5	84.8	3.3	151.5	25
80	PRIME-MoE	0.3395	80.5	94	8.1	59.4	25
90	LSTM	0.0014	90.6	73.8	2.2	260.5	12
90	PRIME-MoE	0.0028	90.6	80.5	2.9	193.6	12

a PRIME-MoE: Postoperative Risk Modeling Expert network.

b Thresholds corresponding to the prespecified sensitivity targets were determined using the validation set and then applied without modification to the corresponding internal test set.

c PPV: positive predictive value.

d FP: false positive.

e FN: false negative.

f INSPIRE: Informative Surgical Patient Dataset for Innovative Research Environment.

At a sensitivity of 90.6% in HUAXI, PRIME-MoE generated 235.1 false positives per 1000 procedures, compared with 308.4 for LSTM, while both models missed 5 deaths. In the INSPIRE test set, at a sensitivity of 80.5%, PRIME-MoE generated 59.4 false positives per 1000 procedures, compared with 151.5 for LSTM. At a sensitivity of 90.6%, the corresponding false-positive burdens were 193.6 and 260.5 per 1000 procedures, respectively. The false-negative counts were identical between the 2 models at each matched-sensitivity operating point.

Compared with LSTM, PRIME-MoE reduced false-positive burden at the matched 80% and 90% sensitivity operating points in both databases. Nevertheless, positive predictive value remained low, particularly in HUAXI. The expanded operating-point analysis (Table S15 in Multimedia Appendix 1) shows why *F*_1_-maximizing thresholds and a fixed threshold of 0.5 are not appropriate universal defaults. Institutions should choose a locally validated sensitivity-alert burden trade-off after recalibration and then monitor false positives, missed deaths, and alert frequency prospectively.

### Cross-Database Generalization

Reciprocal evaluation showed that discrimination was retained better than calibration (Table S2 in Multimedia Appendix 1). The HUAXI-trained model achieved an AUROC of 0.908 and an AUPRC of 0.128 in INSPIRE; the INSPIRE-trained model achieved an AUROC of 0.878 and an AUPRC of 0.081 in HUAXI. Differences in patient characteristics, surgical case mix, outcome prevalence, calendar period, geographic setting, and data collection practices represent a substantial distribution shift. These results indicate shared discriminative signal across the 2 databases rather than universal clinical transportability.

### Calibration

Calibration metrics are summarized in Table 4. In internal evaluation, the Brier score, calibration slope, intercept, and expected calibration error were 0.045, 0.35, –4.06, and 0.054 in INSPIRE and 0.035, 0.40, –4.11, and 0.048 in HUAXI, respectively. Reciprocal evaluations showed similar miscalibration, with slopes of 0.28 and 0.49. Thus, discrimination remained useful, but raw predicted probabilities were not aligned with observed event rates. Reliability diagrams are provided in Figure S2 in Multimedia Appendix 1. These findings support site-specific recalibration before thresholded clinical use.

**Table 4.** Calibration metrics for PRIME-MoE^a^^,^^b^.

Setting	Surgical cases (in-hospital deaths), n	Prevalence (%)	AUROC^c^	AUPRC^d^	Brier (95% CI)	Slope	Intercept	ECE^e^
INSPIRE^f^ internal	19,743 (128)	0.65	0.937	0.222	0.045 (0.042‐0.048)	0.35	−4.06	0.054
HUAXI internal	14,749 (53)	0.36	0.907	0.064	0.035 (0.033‐0.038)	0.40	−4.11	0.048
INSPIRE to HUAXI	14,749 (53)	0.36	0.878	0.081	0.048 (0.046‐0.052)	0.28	−4.63	0.063
HUAXI to INSPIRE	19,743 (128)	0.65	0.908	0.128	0.044 (0.041‐0.047)	0.49	−3.85	0.067

a PRIME-MoE: Postoperative Risk Modeling Expert network.

b Calibration slope and intercept were estimated by regressing the outcome on the logit of predicted probability. Ideal values are 1 and 0, respectively.

c AUROC: area under the receiver operating characteristic curve.

d AUPRC: area under the precision-recall curve.

e ECE: expected calibration error using 10 equal-width bins.

f INSPIRE: Informative Surgical Patient Dataset for Innovative Research Environment.

### Subgroup Analysis

We further conducted exploratory analyses across broad surgical site categories and ASA physical status groups (Tables S3-S6 in Multimedia Appendix 1). Across both databases, PRIME-MoE showed numerically higher or comparable AUROC values relative to LSTM in most surgical subgroups. However, several subgroups contained very few postoperative in-hospital deaths, including central nervous system surgery in HUAXI (2/134) and head and neck surgery in HUAXI (4/647), resulting in wide CIs and potentially unstable comparisons. Unadjusted DeLong *P* values are provided for descriptive purposes, but these analyses were not designed for confirmatory hypothesis testing. Accordingly, interpretation focused on the direction and magnitude of the AUROC differences, CIs, sample sizes, and event counts rather than on isolated *P* values. In HUAXI, the largest numerical difference was observed in cardiovascular surgery, for which PRIME-MoE achieved an AUROC of 81.1 (95% CI 73.1‐89.0), compared with 55.6 (95% CI 41.9‐69.3) for LSTM. In INSPIRE, numerical differences were observed in several categories, including musculoskeletal, cardiovascular, respiratory, and urinary and reproductive surgery. However, because some subgroup event counts were small and multiple comparisons were conducted, these findings should be considered hypothesis-generating rather than evidence of model superiority within individual surgical categories. In the ASA subgroup analyses, PRIME-MoE and LSTM showed comparable discrimination among patients with ASA physical status 1 to 2 in HUAXI, whereas PRIME-MoE had a numerically higher AUROC among patients with ASA physical status 3 to 6. In INSPIRE, PRIME-MoE showed higher AUROC values than LSTM in both ASA groups. These findings likewise represent exploratory assessments of model robustness across broad risk strata. Because the composition of the Other category differed between the source taxonomies of INSPIRE and HUAXI, its subgroup results should not be interpreted as a direct procedure-matched cross-database comparison.

### Computational Profile and Expert Routing

PRIME-MoE with *K*=4 contained 665,029 trainable parameters, compared with 555,775 for the LSTM baseline with hidden size 256 and 196,066 for PRIME-MoE with *K*=1 (Table S12 in Multimedia Appendix 1). Router weights varied significantly by surgical site (INSPIRE: *χ*^2^_24_=822.7; *P*<.001; HUAXI: *χ*^2^_24_=1272.4; *P*<.001). Mean Expert 0 weight was lower in cardiovascular and respiratory cases and higher in urinary and reproductive and skin and soft-tissue cases (Figure 2). Expert 0 was nevertheless the highest-weighted expert for 18,815/19,743 (95.3%) of INSPIRE and 10,565/14,749 (71.6%) of HUAXI cases. We therefore interpret the pattern as a shared generalist expert supplemented by context-dependent residual experts, not as evidence of fully disjoint procedure-specific specialists.

**Figure 2.**
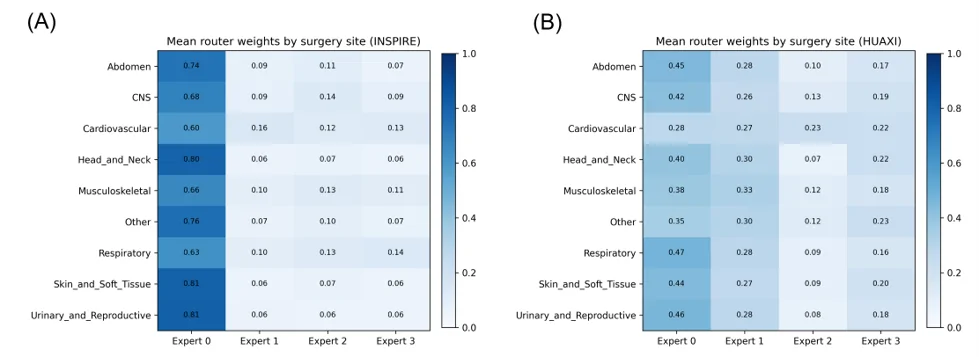
Mean Postoperative Risk Modeling Expert network (PRIME-MoE) router weights by surgical site in (A) INSPIRE (Informative Surgical Patient Dataset for Innovative Research Environment) and (B) HUAXI. Expert 0 remained the highest-weighted expert for most cases, while secondary-expert participation varied by surgical context. Surgical site was associated with the dominant expert in both databases (*P*<.001). CNS: central nervous system.

### Feature Importance Analysis

We used the IG method to quantify the contribution of both static and dynamic variables to postoperative in-hospital mortality predictions generated by the PRIME-MoE model. Overall, dynamic intraoperative features received higher attribution values than most static features. Heart rate had the highest average IG attribution in both databases, indicating that the model relied strongly on heart-rate information relative to the selected baseline (Figure 3). Other dynamic variables with relatively high attributions included arterial diastolic blood pressure, oxygen concentration, arterial blood gas parameters, peak inspiratory pressure, end-tidal CO_₂_, and bispectral index. These attributions indicate associations with model predictions but do not distinguish physiological severity from the effects of treatment and intraoperative management. Static features showed comparatively lower but nonnegligible contributions. Variables such as age, ASA classification, emergency status, and selected laboratory indicators provided important baseline risk context, complementing the time-varying physiological signals rather than dominating the prediction. Figure 4 illustrates a representative case predicted by PRIME-MoE. The patient was a 70-year-old female undergoing respiratory surgery, for whom the model assigned a high predicted risk of in-hospital mortality. The IG heatmaps showed progressively increasing attribution values for several dynamic inputs toward the later intraoperative period. These patterns indicate increasing model reliance on the corresponding inputs but should not be interpreted as direct evidence of clinical deterioration or causal effects. Meanwhile, the MoE weights revealed a highly concentrated routing pattern, with Expert 0 receiving the majority of the activation weight, while the remaining experts contributed marginally. This case illustrates how PRIME-MoE combines expert routing with temporal feature attribution to provide a model-level explanation of an individual prediction. The attribution results are descriptive and should not be used independently to guide treatment decisions.

**Figure 3.**
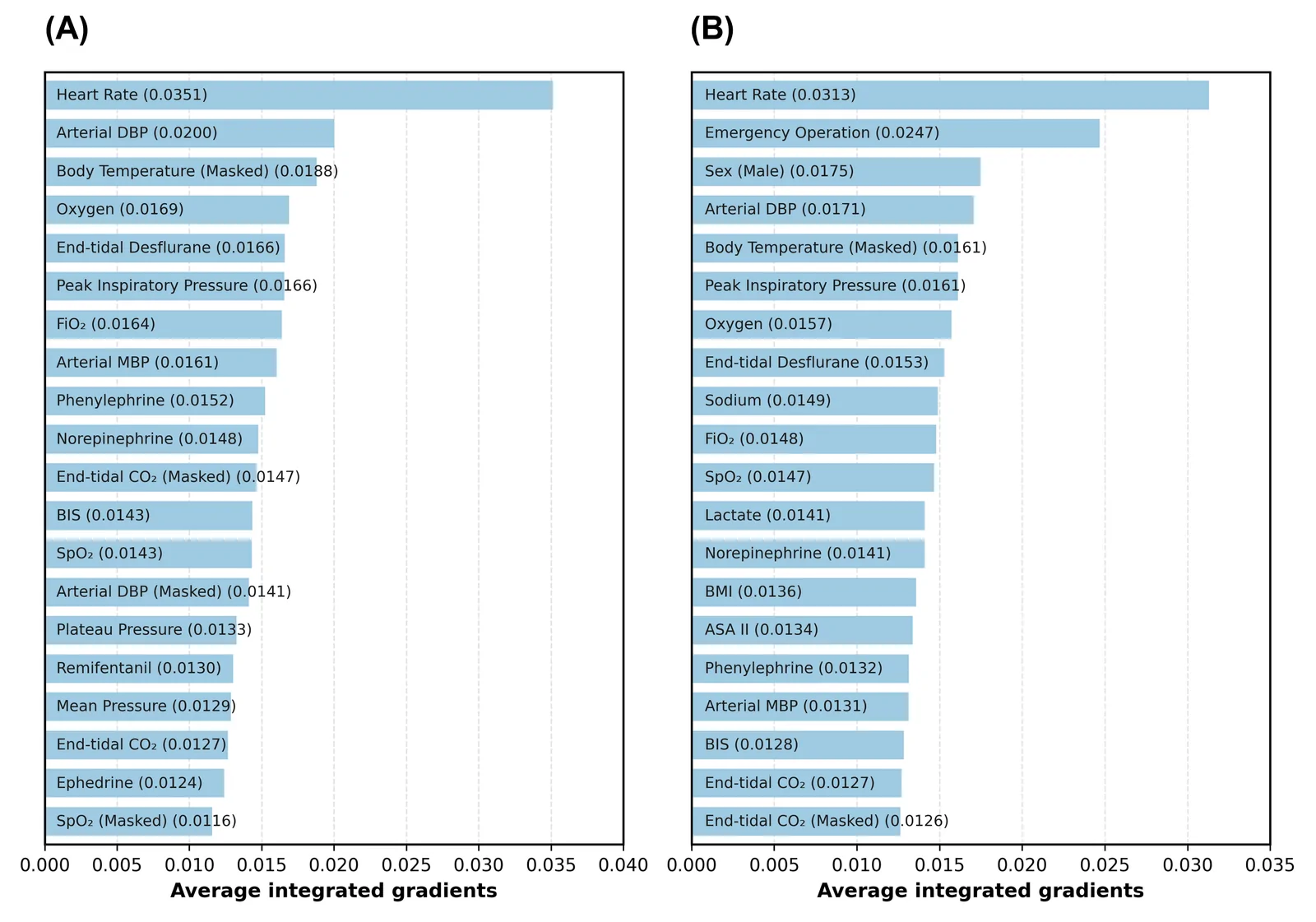
(A) Top 20 integrated gradients attributions of the Postoperative Risk Modeling Expert network (PRIME-MoE) model for predicting postoperative in-hospital mortality risk in the INSPIRE (Informative Surgical Patient Dataset for Innovative Research Environment) database. (B) Top 20 integrated gradients attributions in the HUAXI database. Integrated gradients attributions quantify model dependence relative to the selected baseline and should not be interpreted as causal effects. Intervention-sensitive variables may reflect underlying physiological instability, treatment response, or clinical management intensity. Categorical variables were represented using full one-hot encoding. For these variables, the zero baseline represents the absence of an individual category indicator rather than an actual patient category; therefore, category-specific attributions should not be interpreted as comparisons with another category. ASA: American Society of Anesthesiologists; BIS: bispectral index; DBP: diastolic blood pressure; FiO_2_: fraction of inspired oxygen; MBP: mean blood pressure; SpO_2_: peripheral oxygen saturation.

**Figure 4.**
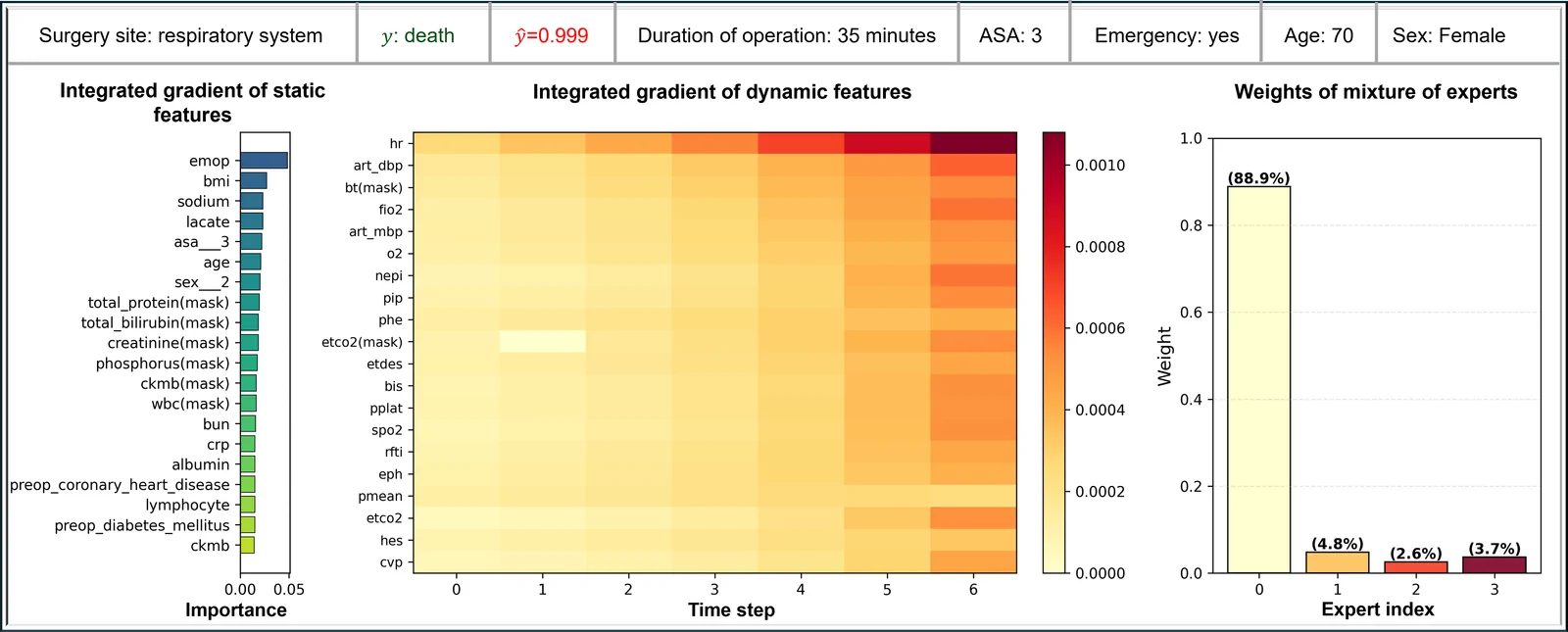
Representative case of postoperative in-hospital mortality risk prediction by Postoperative Risk Modeling Expert network (PRIME-MoE), showing integrated gradients attributions for static and dynamic inputs and the corresponding mixture-of-experts weights. Integrated gradients attributions describe model dependence relative to the selected baseline and should not be interpreted as causal effects, treatment effects, or evidence that modifying a highly attributed variable would change mortality risk. For 1-hot encoded categorical inputs, the zero baseline represents the absence of each category indicator and does not constitute a clinically realizable patient profile. The displayed categorical attributions therefore reflect the contribution of activated indicators relative to this mathematical baseline rather than category-to-category comparisons. ASA: American Society of Anesthesiologists; BIS: bispectral index; BMI: body mass index; BUN: blood urea nitrogen; CO₂: carbon dioxide; CRP: C-reactive protein; CVP: central venous pressure; DBP: diastolic blood pressure; FiO_2_: fraction of inspired oxygen; MBP: mean blood pressure; SpO_2_: peripheral oxygen saturation; WBC: white blood cell.

## Discussion

### Principal Findings

We developed PRIME-MoE for postoperative in-hospital mortality prediction using 2 large perioperative databases. Internal AUROCs were 0.938 in INSPIRE and 0.907 in HUAXI. Reciprocal evaluation retained discrimination, but calibration slopes of 0.28‐0.49 showed that probabilities were not directly transportable. Scoring 30 minutes before the recorded end of surgery reduced AUROC by only 0.006 in INSPIRE and 0.005 in HUAXI, and median neural-network inference time was 11.4 ms per case. These findings support further evaluation of PRIME-MoE as a near-end-of-surgery triage aid, with local recalibration and prospective workflow validation before deployment.

### Interpretation and Comparison With Prior Work

Across the INSPIRE and HUAXI cohorts, PRIME-MoE achieved higher AUROC and AUPRC values than the evaluated logistic regression, XGBoost, and LSTM models. Each temporal expert was a 1-layer unidirectional LSTM, and static perioperative context generated dense softmax weights over all experts. This architecture allowed shared temporal representations to be combined according to surgical context, but the present analyses do not establish that gating is superior to simple context concatenation. A direct retrained ablation remains necessary.

Previous clinical MoE studies used multiple temporal experts to represent heterogeneous patient trajectories [28-30]. PRIME-MoE differs in that it uses static perioperative context to assign patient-specific dense weights to LSTM experts, each of processes the complete intraoperative sequence. Its contribution is the application and reciprocal evaluation of this context-informed routing strategy for postoperative in-hospital mortality prediction.

### Cross-Database Generalization and Subgroup Performance

Reciprocal evaluation indicates that PRIME-MoE captured shared discriminative signal across INSPIRE and HUAXI despite differences in patient mix, surgical categories, outcome prevalence, calendar period, and institutional practice. The HUAXI-trained model achieved an AUROC of 0.908 in INSPIRE, whereas the INSPIRE-trained model achieved an AUROC of 0.878 in HUAXI. These results should not be interpreted as universal transportability.

Calibration results sharpen this limitation. Slopes substantially below 1 and large negative intercepts show that raw probability estimates were not transferable, even when AUROC remained useful. The low HUAXI AUPRC (0.064 internally) also indicates a high false-positive burden in a lower-prevalence setting. Reciprocal validation therefore supports shared signal, not plug-and-play clinical deployment. New sites should recalibrate the model and select operating points on local validation data.

### Expert Activation Patterns

Router weights were associated with surgical site in both databases, but one expert remained dominant for most patients. The lower Expert 0 weights in cardiovascular and respiratory procedures and greater participation of secondary experts suggest context-dependent routing. However, this pattern does not prove that individual experts represent distinct physiological mechanisms. We therefore describe the result as a shared generalist expert with residual context-dependent experts and treat a direct concatenation-versus-gating ablation as future work.

### Clinical and Methodological Implications

PRIME-MoE contained about 20% more trainable parameters than the LSTM baseline used in the paper (665,029 vs 555,775). Synthetic GPU inference at sequence length 40 was approximately 7.2 ms per case for PRIME-MoE and 1.8 ms for the LSTM baseline. Although PRIME-MoE was slower, real-data scoring remained in the millisecond range. Training wall-clock time was not recorded prospectively and could not be reconstructed reliably; this is reported transparently rather than estimated after the fact.

PRIME-MoE is positioned as an end-of-surgery or near-end postoperative triage aid, not an intraoperative closed-loop controller. A prospective score could be generated when surgery end is anticipated, provided that the preoperative features and intraoperative stream up to that time are available. The 30-minute truncation analysis suggests that much of the discriminative information is available before formal surgery end, but it does not replace prospective testing of data availability and clinical workflow.

Model evaluation required only milliseconds per case in the GPU benchmarks; end-to-end latency will depend on data extraction, resampling, and integration with the anesthesia information system. Scores should support clinician review of PACU or higher-acuity disposition and should not independently mandate ICU admission or another intervention.

No universal probability threshold is supported by these data. Institutions should choose a sensitivity target based on the intended use and acceptable alerts per 1000 procedures; recalibrate probabilities locally; freeze the selected threshold for evaluation; and monitor positive predictive value, false positives, and missed deaths after deployment.

At matched sensitivity, PRIME-MoE produced fewer false positives than LSTM, but the alert burden increased sharply at higher sensitivity and positive predictive value remained low. A continuous or tiered risk display may therefore be more appropriate than an isolated binary alarm. Prospective studies should measure alert frequency, clinician response, changes in postoperative disposition, and patient outcomes.

### Interpreting Feature Attributions for Clinical Readers

GI attributions quantify how each input contributes to a model prediction relative to the selected baseline. They describe associations with the model output, not causal effects on postoperative death. This distinction is essential for perioperative readers.

Highly attributed variables such as heart rate, arterial pressure, oxygenation measures, and vasoactive drug exposure are also treatment targets or indicators of treatment intensity. A vasopressor may receive high attribution because it marks hemodynamic instability that prompted treatment, not because the drug caused mortality. Blood-pressure and heart-rate patterns can likewise reflect both patient state and the response to clinical management.

IG can be used to audit whether the model relies on clinically plausible signals, explain why an individual score is elevated, and generate hypotheses for future studies. It should not be used to rank independent causal risk factors, justify changing a drug solely because it has high attribution, or replace clinical judgment about reversible intraoperative physiology.

Complementary tools can provide different views of model behavior. Shapley additive explanations (SHAP) offers alternative additive explanations, and router-weight visualizations summarize which temporal experts receive weight for a given surgical context. Counterfactual or treatment-aware models would be required for causal questions. Until such approaches are validated prospectively, clinical displays should include an explicit noncausal disclaimer. Related work has incorporated human expertise into MoE models and developed interpretable representations for multivariate medical time series [39-41]. These approaches are complementary to IG and may support future comparisons of interpretability methods in perioperative prediction.

### Fairness Considerations

Interpretability does not eliminate bias. PRIME-MoE may reproduce disparities in historical care or perform less reliably in rare surgical conditions and underrepresented groups. Prospective evaluation should therefore examine calibration, error rates, and clinical consequences across relevant subgroups. Clinical deployment would also require explicit governance for patient privacy, clinician oversight, accountability, and appropriate limits on automated decision-making [42,43].

### Limitations

This study has several limitations. First, it was retrospective and did not measure streaming-data latency, system integration, or effects on PACU or ICU decisions. Second, postoperative in-hospital mortality may be influenced by discharge practice and does not capture 30- or 90-day mortality. Third, both databases came from large East Asian academic hospitals, so performance in other countries, community hospitals, and different surgical case mixes is unknown. Fourth, raw probability calibration was suboptimal and requires site-specific recalibration before thresholded use. Fifth, expert routing was associated with surgical context but was dominated by a shared expert; a retrained ablation against simple feature concatenation remains necessary. Sixth, regular-grid completion may introduce imputation-related artifacts despite observation masks, and removal of time points without source observations can shorten usable sequences. Missingness-aware encoders such as GRU-D [44] are alternatives for future study. Seventh, several subgroup comparisons contained few or no events; their CIS and unadjusted DeLong tests are exploratory. Finally, the 30-minute analysis reused end-of-surgery-trained weights and retained at least 1 bin for short operations; a prospective model trained and evaluated at a prespecified earlier trigger may perform differently.

### Conclusions

In conclusion, PRIME-MoE combined static preoperative context with intraoperative time-series data and retained useful discrimination across 2 heterogeneous perioperative databases. Scoring 30 minutes before the recorded end of surgery produced only small AUROC reductions, and neural-network inference was fast. However, poor calibration and low positive predictive value in the lower-prevalence cohort preclude direct deployment. Prospective external validation, local recalibration, threshold selection, and workflow evaluation are required before clinical use.

## Supplementary material

10.2196/92856 Multimedia Appendix 1 Supplementary figures and tables detailing cohort characteristics, data preprocessing, model development, performance evaluation, calibration, and sensitivity analyses.

10.2196/92856 Checklist 1 TRIPOD+AI checklist.

## Abbreviations

ASA: American Society of Anesthesiologists
AUPRC: area under the precision-recall curve
AUROC: area under the receiver operating characteristic curve
ECE: expected calibration error
EHR: electronic health record
EMR-LIP: Electronic Medical Record-Longitudinal Irregular Preprocessing
GPU: graphics processing unit
GRU: Gated Recurrent Unit
ICU: intensive care unit
IG: integrated gradients
INSPIRE: Informative Surgical Patient Dataset for Innovative Research Environment
LSTM: long short-term memory
MLP: multilayer perceptron
MoE: Mixture of Experts
PACU: postanesthesia care unit
PRIME-MoE: Postoperative Risk Modeling Expert network
SHAP: Shapley additive explanations
TRIPOD+AI: Transparent Reporting of a multivariable prediction model for Individual Prognosis Or Diagnosis + Artificial Intelligence
XGBoost: extreme gradient boosting
