# Supplementary material for: Development and Validation of a Mixture-of-Experts–Based Model for Postoperative In-Hospital Mortality Risk Assessment: Multicenter Retrospective Study
Source: J Med Internet Res. 2026 Nov 4;28:e92856. doi: 10.2196/92856 (PMC13734703; doi:10.2196/92856)
Supplement: Supplementary file 2 [file jmir-v28-e92856-s001.docx]

**Multimedia Appendix 1**

**Detailed Introduction of the PRIME-MoE Model**

**Implemented PRIME-MoE Architecture**

PRIME-MoE integrates a static preoperative vector with a variable-length intraoperative sequence. The released mortality checkpoints use four parallel one-layer unidirectional LSTM experts. Each expert receives the 143-dimensional dynamic sequence, including observation masks, and produces a 128-dimensional temporal embedding.

**Router and Static Encoders**

The 111-dimensional static vector is processed by a two-layer MLP with hidden size 64. A separate router MLP with hidden size 32 maps the same static vector to four softmax weights. Routing is dense: every expert contributes to the weighted temporal embedding. The model does not apply top-k selection or sparse activation.

**Prediction Head and Training**

The weighted temporal embedding is concatenated with the static embedding and passed through a prediction MLP with hidden size 128 and a sigmoid output. The final checkpoints contain no dropout layers. Models were trained with weighted binary cross-entropy and Adam (learning rate 0.001; weight decay 0.001), batch size 150, and early stopping based on validation AUROC.

**Surgery Adaptive Definition**

Surgery-adaptive refers to patient-specific weighting of shared temporal experts using static surgical context, including surgical category and ASA class. It does not mean that a separate model is trained for every surgical group, and the routing weights should not be interpreted as proof that an expert encodes a unique physiological mechanism.

**Supplemental Figures**

**Figure S1**. Sensitivity analysis results for the number of experts (K).

**Supplemental Tables**

**Table S1**. Baseline characteristics of different datasets in the INSPIRE and HUAXI databases

|  | Train | Test | Valid |
| --- | --- | --- | --- |
| HUAXI |  |  |  |
| N, operations | 51978 | 14851 | 7426 |
| Age, years | 60.24 ± 15.13 | 59.98 ± 15.21 | 60.24 ± 14.94 |
| Gender |  |  |  |
| Female | 23356 (44.94%) | 6779 (45.65%) | 3268 (44.01%) |
| Male | 28621 (55.06%) | 8072 (54.35%) | 4158 (55.99%) |
| BMI, kg/m^2^ | 23.39 ± 3.47 | 23.43 ± 3.51 | 23.34 ± 3.47 |
| ASA grade |  |  |  |
| 1-2 | 33769 (64.97%) | 9673 (65.13%) | 4891 (65.86%) |
| 3-4 | 18180 (34.98%) | 5176 (34.85%) | 2532 (34.1%) |
| >=5 | 29 (0.06%) | 2 (0.01%) | 3 (0.04%) |
| Emergency |  |  |  |
| No | 49198 (94.65%) | 14026 (94.44%) | 7016 (94.48%) |
| Yes | 2780 (5.35%) | 825 (5.56%) | 410 (5.52%) |
| Surgery sites |  |  |  |
| Abdomen | 26551 (51.08%) | 7502 (50.52%) | 3779 (50.89%) |
| Cardiovascular | 1439 (2.77%) | 409 (2.75%) | 194 (2.61%) |
| CNS | 479 (0.92%) | 125 (0.84%) | 76 (1.02%) |
| Head_and_Neck | 2302 (4.43%) | 642 (4.32%) | 316 (4.26%) |
| Musculoskeletal | 7913 (15.22%) | 2346 (15.8%) | 1120 (15.08%) |
| Other | 686 (1.32%) | 202 (1.36%) | 95 (1.28%) |
| Respiratory | 6347 (12.21%) | 1854 (12.48%) | 946 (12.74%) |
| Skin_and_Soft_Tissue | 1931 (3.72%) | 568 (3.82%) | 269 (3.62%) |
| Urinary_and_Reproductive | 4330 (8.33%) | 1203 (8.1%) | 631 (8.5%) |
| Duration of the surgery, minutes | 137.02 ± 95.52 | 136.27 ± 96.77 | 135.46 ± 94.81 |
| Duration of anesthesia, minutes | 203.64 ± 112.82 | 202.88 ± 113.86 | 202.06 ± 112.95 |
| Postoperative in-hospital death |  |  |  |
| Survived | 51781 (99.62%) | 14781 (99.53%) | 7408 (99.76%) |
| Died | 197 (0.38%) | 70 (0.47%) | 18 (0.24%) |
| INSPIRE |  |  |  |
| N, operations | 69865 | 19743 | 10127 |
| Age, years | 54.65 ± 15.67 | 54.41 ± 15.75 | 54.73 ± 15.66 |
| Gender |  |  |  |
| Female | 38148 (54.6%) | 11181 (56.63%) | 5528 (54.59%) |
| Male | 31717 (45.4%) | 8562 (43.37%) | 4599 (45.41%) |
| BMI | 23.66 ± 3.47 | 23.63 ± 3.45 | 23.63 ± 3.49 |
| ASA |  |  |  |
| 1-2 | 60960 (89.69%) | 17276 (90.07%) | 8809 (89.43%) |
| 3-4 | 6941 (10.21%) | 1875 (9.78%) | 1035 (10.51%) |
| >=5 | 70 (0.1%) | 30 (0.16%) | 6 (0.06%) |
| Emergency |  |  |  |
| No | 62897 (90.03%) | 17769 (90%) | 9095 (89.81%) |
| Yes | 6968 (9.97%) | 1974 (10%) | 1032 (10.19%) |
| Surgery sites |  |  |  |
| Abdomen | 15352 (21.97%) | 4369 (22.13%) | 2116 (20.89%) |
| Cardiovascular | 1961 (2.81%) | 529 (2.68%) | 287 (2.83%) |
| CNS | 3257 (4.66%) | 913 (4.62%) | 511 (5.05%) |
| Head_and_Neck | 11426 (16.35%) | 3204 (16.23%) | 1651 (16.3%) |
| Musculoskeletal | 6602 (9.45%) | 1914 (9.69%) | 1017 (10.04%) |
| Other | 8211 (11.75%) | 2301 (11.65%) | 1213 (11.98%) |
| Respiratory | 3191 (4.57%) | 883 (4.47%) | 435 (4.3%) |
| Skin_and_Soft_Tissue | 9234 (13.22%) | 2611 (13.22%) | 1321 (13.04%) |
| Urinary_and_Reproductive | 10631 (15.22%) | 3019 (15.29%) | 1576 (15.56%) |
| Duration of the surgery, minutes | 136.74 ± 111.21 | 135.36 ± 110.94 | 137.52 ± 110.84 |
| Duration of anesthesia, minutes | 174.19 ± 121.15 | 172.6 ± 119.73 | 175.92 ± 121.67 |
| Postoperative in-hospital death |  |  |  |
| Survived | 69468 (99.43%) | 19615 (99.35%) | 10075 (99.49%) |
| Died | 397 (0.57%) | 128 (0.65%) | 52 (0.51%) |

**Table S2**. Summary of cross-database generalization performance of the PRIME-MoE model on the INSPIRE and HUAXI databases

|  | AUROC | AUPRC | Accuracy | Recall | Precision | F1 |
| --- | --- | --- | --- | --- | --- | --- |
| HUAXI to HUAXI | 90.7 (86.9, 94.7) | 6.4 (4.4, 12.7) | 99.3 (98.2, 99.5) | 17.0 (11.8, 44.5) | 13.4 (6.4, 24.7) | 15.0 (10.5, 26.4) |
| HUAXI to INSPIRE | 90.8 (88.6, 93.1) | 12.8 (9.5, 18.5) | 98.9 (98.8, 99.2) | 25.0 (16.6, 34.6) | 21.1 (15.4, 29.0) | 22.9 (16.8, 29.6) |
| INSPIRE to HUAXI | 89.4（84.5, 93.0） | 8.4（3.1, 14.9） | 99.2 (97.1, 99.7) | 18.9 (6.5, 37.2) | 11.0 (4.1, 36.0) | 13.9 (6.8, 21.2) |
| INSPIRE to INSPIRE | 93.7 (91.3, 95.0) | 22.2 (17.5, 31.2) | 98.8 (98.7, 99.2) | 43.8 (31.5, 50.4) | 25.8 (21.2, 41.5) | 32.5 (27.7, 39.4) |

**Table S3**. Performance comparison between PRIME-MoE and LSTM in different surgical site subgroups on the HUAXI database.

| Surgery site | In-hospital mortality | PRIME-MoE | LSTM | Delong Z | Unadjusted *P* |
| --- | --- | --- | --- | --- | --- |
| Abdomen | 0.4% (27/7503) | 86.4(79.0, 93.7) | 85.7(78.9, 92.6) | 0.538 | 0.59 |
| Cardiovascular | 2.2% (9/416) | 81.1(73.1, 89.0) | 55.6(41.9, 69.3) | 3.484 | <0.001 |
| Central nervous system | 1.5% (2/134) | 93.6(86.0, 100.0) | 98.5(96.1, 100.0) | -1.08 | 0.28 |
| Head and neck | 0.6% (4/647) | 96.5(91.6, 100.0) | 91.5(79.9, 100.0) | 1.433 | 0.152 |
| Musculoskeletal | 0.1% (3/2295) | 90.1(81.9, 98.2) | 86.1(72.4, 99.7) | 0.835 | 0.404 |
| Other | 2.8% (5/179) | 97.8(94.6, 100.0) | 97.7(95.3, 100.0) | 0.058 | 0.954 |
| Respiratory | 0.2% (3/1833) | 99.6(99.2, 100.0) | 96.4(92.8, 100.0) | 1.95 | 0.051 |
| Skin and soft tissue | 0.0% (0/487) | - | - | - | - |
| Urinary and reproductive | 0.0% (0/1255) | - | - | - | - |

P values were derived from exploratory pairwise DeLong tests comparing the AUROCs of PRIME-MoE and LSTM within each surgical subgroup. No adjustment for multiple comparisons was applied. Because several subgroups contained few outcome events, the P values should be interpreted together with the subgroup sample sizes, event counts, effect estimates, and 95% confidence intervals and should not be considered confirmatory.

**Table S4**. Performance comparison between PRIME-MoE and LSTM in different surgical site subgroups on the INSPIRE database

| Surgery site | In-hospital mortality | PRIME-MoE | LSTM | Delong Z | Unadjusted *P* |
| --- | --- | --- | --- | --- | --- |
| Abdomen | 0.4% (18/4369) | 93.0(84.9, 100.0) | 89.1(79.7, 98.5) | 2.556 | 0.011 |
| Cardiovascular | 1.1% (6/529) | 85.3(63.3, 100.0) | 56.7(44.2, 69.2) | 2.658 | 0.008 |
| Central nervous system | 1.6% (15/913) | 86.2(77.8, 94.5) | 84.2(75.5, 93.0) | 0.851 | 0.395 |
| Head and neck | 0.3% (10/3204) | 91.2(77.5, 100.0) | 91.5(81.8, 100.0) | -0.112 | 0.911 |
| Musculoskeletal | 0.8% (15/1914) | 88.9(79.4, 98.4) | 84.4(74.4, 94.4) | 3.535 | <0.001 |
| Other | 0.8% (19/2301) | 95.7(92.2, 99.2) | 93.8(89.7, 97.8) | 1.509 | 0.131 |
| Respiratory | 1.5% (13/883) | 97.7(96.1, 99.3) | 88.5(77.8, 99.3) | 1.906 | 0.057 |
| Skin and soft tissue | 0.5% (12/2611) | 96.3(93.2, 99.4) | 92.8(87.6, 98.0) | 2.68 | 0.007 |
| Urinary and reproductive | 0.7% (20/3019) | 99.3(98.8, 99.8) | 96.2(93.7, 98.7) | 2.619 | 0.009 |

P values were derived from exploratory pairwise DeLong tests comparing the AUROCs of PRIME-MoE and LSTM within each surgical subgroup. No adjustment for multiple comparisons was applied. Because several subgroups contained few outcome events, the P values should be interpreted together with the subgroup sample sizes, event counts, effect estimates, and 95% confidence intervals and should not be considered confirmatory.

**Table S5**. Performance comparison between PRIME-MoE and LSTM in different ASA subgroups on the HUAXI database

| ASA group | In-hospital mortality | PRIME-MoE | LSTM | Delong Z | *P* |
| --- | --- | --- | --- | --- | --- |
| 1-2 | 0.1% (6/9611) | 75.1(57.8, 92.4) | 76.4(59.8, 93.1) | -0.955 | 0.339 |
| 3-6 | 0.9% (47/5138) | 88.3(83.8, 92.9) | 84.0(79.1, 88.8) | 2.856 | 0.004 |

**Table S6**. Performance comparison between PRIME-MoE and LSTM in different ASA subgroups on the INSPIRE database

| ASA group | In-hospital mortality | PRIME-MoE | LSTM | Delong Z | *P* |
| --- | --- | --- | --- | --- | --- |
| 1-2 | 0.2% (32/17276) | 88.4(81.2, 95.7) | 83.1(76.2, 89.9) | 3.613 | <0.001 |
| 3-6 | 4.1% (78/1905) | 88.1(84.5, 91.8) | 83.5(79.0, 88.0) | 3.096 | 0.002 |

**Table S7**. List of features used as inputs to the PRIME-MoE model

|  | Value type | Time type | Unit | Range | HUAXI  missingness (%) | INSPIRE missingness (%) |
| --- | --- | --- | --- | --- | --- | --- |
| Demographics and Preoperative Characteristics | | | | |  |  |
| Age | num | static | years | 0-120 | 1.43 | 0 |
| Sex | cat | static | — | male/female | 1.43 | 0 |
| Weight | num | static | kg | 0.5-350 | 1.65 | 1.12 |
| Height | num | static | cm | 30-250 | 1.81 | 0.6 |
| Body mass index | num | static | kg/m² | 5.0-100.0 | 1.84 | 1.37 |
| ASA physical status | cat | static | — | 1-6 | 1.49 | 2.71 |
| Emergency operation | bin | static | — | 0-1 | 1.43 | 0 |
| Surgery site | cat | static | — | valid categories | 1.43 | 0 |
| Preoperative hypertension | bin | static | — | 0-1 | 1.43 | 0 |
| Preoperative coronary heart disease | bin | static | — | 0-1 | 1.43 | 0 |
| Preoperative heart failure | bin | static | — | 0-1 | 1.43 | 0 |
| Preoperative atrial fibrillation | bin | static | — | 0-1 | 1.43 | 0 |
| Preoperative arrhythmia | bin | static | — | 0-1 | 1.43 | 0 |
| Preoperative diabetes mellitus | bin | static | — | 0-1 | 1.43 | 0 |
| Preoperative cerebral infarction | bin | static | — | 0-1 | 1.43 | 0 |
| Preoperative transient ischemic attack | bin | static | — | 0-1 | 1.43 | 0 |
| Preoperative chronic obstructive pulmonary disease | bin | static | — | 0-1 | 1.43 | 0 |
| Preoperative asthma | bin | static | — | 0-1 | 1.43 | 0 |
| Preoperative acute respiratory infection | bin | static | — | 0-1 | 1.43 | 0 |
| Preoperative chronic kidney disease | bin | static | — | 0-1 | 1.43 | 0 |
| Preoperative chronic hepatitis | bin | static | — | 0-1 | 1.43 | 0 |
| Preoperative liver disease | bin | static | — | 0-1 | 1.43 | 0 |
| Preoperative gastroesophageal reflux disease | bin | static | — | 0-1 | 1.43 | 0 |
| Preoperative anemia | bin | static | — | 0-1 | 1.43 | 0 |
| Preoperative thyroid disorder | bin | static | — | 0-1 | 1.43 | 0 |
| Operation duration | num | static | minutes | 0-3000 | 2.21 | 0 |
| Anesthesia duration | num | static | minutes | 0-3600 | 2.21 | 0 |
| Laboratory and Blood Gas Parameters (Last Measurement Before Surgery) | | | | |  |  |
| Base excess | num | static | mmol/L | -40-40 | 90.4 | 71.93 |
| Bicarbonate | num | static | mmol/L | 0-80 | 90.4 | 71.05 |
| Partial pressure of arterial carbon dioxide | num | static | mmHg | 5-250 | 90.39 | 81.68 |
| Partial pressure of arterial oxygen | num | static | mmHg | 0-700 | 90.41 | 81.68 |
| Blood pH | num | static | — | 6.5-8.0 | 51.49 | 81.66 |
| Arterial oxygen saturation | num | static | % | 0-100 | 90.41 | 71.05 |
| Hemoglobin | num | static | g/dL | 1-25 | 2.69 | 31.22 |
| Hematocrit | num | static | % | 3-75 | 2.69 | 31.12 |
| Lymphocyte count | num | static | ×10⁹/L | 0-100 | 2.75 | 37.22 |
| Platelet count | num | static | ×10⁹/L | 0-2000 | 2.7 | 31.61 |
| White blood cell count | num | static | ×10⁹/L | 0-500 | 2.38 | 31.38 |
| Albumin | num | static | g/dL | 0.5-8.0 | 2.41 | 40.6 |
| Alkaline phosphatase | num | static | U/L | 0-5000 | 2.4 | 40.73 |
| Alanine aminotransferase | num | static | U/L | 0-20000 | 2.42 | 40.19 |
| Aspartate aminotransferase | num | static | U/L | 0-20000 | 2.4 | 40.12 |
| Blood urea nitrogen | num | static | mmol/L | 0.5-80 | 3.04 | 37.76 |
| Calcium | num | static | mmol/L | 0.5-5.0 | 2.42 | 31.07 |
| Chloride | num | static | mmol/L | 50-140 | 3.31 | 24.14 |
| Creatine kinase | num | static | U/L | 0-100000 | 2.37 | 94.56 |
| Creatinine | num | static | μmol/L | 5-3000 | 2.45 | 36.82 |
| Glucose | num | static | mmol/L | 0.5-60 | 90.16 | 45.7 |
| Lactate | num | static | mmol/L | 0-30 | 3.05 | 98.04 |
| Phosphorus | num | static | mmol/L | 0.1-6.0 | 2.39 | 32.72 |
| Potassium | num | static | mmol/L | 1.0-10.0 | 2.39 | 23.82 |
| Sodium | num | static | mmol/L | 90-190 | 2.4 | 24.08 |
| Total bilirubin | num | static | μmol/L | 0-1000 | 2.39 | 42.39 |
| Total protein | num | static | g/dL | 1.0-12.0 | 4.65 | 40.5 |
| Activated partial thromboplastin time | num | static | s | 5-300 | 72.51 | 45 |
| D-dimer | num | static | mg/L | 0-100 | 4.66 | 99.95 |
| Fibrinogen | num | static | g/L | 0-15 | 4.61 | 51.63 |
| Prothrombin time international normalized ratio | num | static | ratio | 0.5-20 | 79.56 | 44.6 |
| Creatine kinase MB | num | static | ng/mL | 0-1000 | 77.33 | 94.75 |
| C-reactive protein | num | static | mg/L | 0-500 | 87.11 | 57.21 |
| Hemoglobin A1c | num | static | % | 3-20 | 78.51 | 89.11 |
| Troponin T | num | static | ng/mL | 0-100 | 90.4 | 99.99 |
| Intraoperative Physiologic Monitoring | | | | |  |  |
| Air flow | num | dynamic | L/min | 0-20 | 98.14 | 97.98 |
| End-tidal desflurane concentration | num | dynamic | % | 0-18 | 79.77 | 99 |
| End-tidal gas concentration | num | dynamic | % | 0-100 | 98.07 | 97.81 |
| End-tidal isoflurane concentration | num | dynamic | % | 0-5 | 99.88 | 99.89 |
| End-tidal sevoflurane concentration | num | dynamic | % | 0-8 | 47.52 | 99.76 |
| Fraction of inspired oxygen | num | dynamic | % | 0-100 | 74.01 | 14.37 |
| Nitrous oxide concentration | num | dynamic | % | 0-80 | 99.94 | 99.94 |
| Oxygen flow | num | dynamic | L/min | 0-20 | 99.34 | 99.27 |
| Arterial diastolic blood pressure | num | dynamic | mmHg | 0-200 | 39.44 | 36.99 |
| Arterial mean blood pressure | num | dynamic | mmHg | 0-250 | 40.49 | 39.11 |
| Arterial systolic blood pressure | num | dynamic | mmHg | 0-300 | 40.24 | 39.1 |
| Noninvasive diastolic blood pressure | num | dynamic | mmHg | 0-200 | 3.16 | 4.33 |
| Noninvasive mean blood pressure | num | dynamic | mmHg | 0-250 | 4 | 7.7 |
| Noninvasive systolic blood pressure | num | dynamic | mmHg | 0-300 | 3.08 | 6.95 |
| Bispectral index | num | dynamic | — | 0-100 | 75.55 | 80.68 |
| Body temperature | num | dynamic | °C | 20-45 | 94.57 | 10.02 |
| Cerebral regional oxygen saturation | num | dynamic | % | 0-100 | 99.92 | 99.92 |
| Cardiac index | num | dynamic | L/min/m² | 0-15 | 97.88 | 97.56 |
| Central venous pressure | num | dynamic | mmHg | -10-50 | 96.9 | 71.7 |
| Heart rate | num | dynamic | bpm | 0-300 | 2.94 | 2.38 |
| Minute ventilation | num | dynamic | L/min | 0-30 | 64.11 | 10.73 |
| Pulmonary artery diastolic pressure | num | dynamic | mmHg | 0-80 | 95.52 | 95.18 |
| Pulmonary artery mean pressure | num | dynamic | mmHg | 0-100 | 95.29 | 94.82 |
| Pulmonary artery systolic pressure | num | dynamic | mmHg | 0-150 | 95.95 | 95.69 |
| Peripheral oxygen saturation | num | dynamic | % | 0-100 | 1.9 | 2.26 |
| ST segment in lead I | num | dynamic | mV | -1.0-1.0 | 99.75 | 93.84 |
| ST segment in lead II | num | dynamic | mV | -1.0-1.0 | 98.03 | 82.48 |
| ST segment in lead III | num | dynamic | mV | -1.0-1.0 | 96.25 | 95.79 |
| ST segment in lead V5 | num | dynamic | mV | -1.0-1.0 | 96.98 | 96.49 |
| Stroke volume index | num | dynamic | mL/m² | 0-150 | 99.58 | 99.57 |
| End-tidal carbon dioxide | num | dynamic | mmHg | 0-120 | 5.91 | 8.96 |
| Positive end-expiratory pressure | num | dynamic | cmH₂O | 0-40 | 68.26 | 34.97 |
| Peak inspiratory pressure | num | dynamic | cmH₂O | 0-80 | 64.44 | 9.81 |
| Mean airway pressure | num | dynamic | cmH₂O | 0-60 | 64.69 | 40.31 |
| Plateau pressure | num | dynamic | cmH₂O | 0-80 | 67.95 | 51.84 |
| Respiratory rate | num | dynamic | breaths/min | 0-80 | 4.38 | 6.89 |
| Tidal volume | num | dynamic | mL | 0-2000 | 64.45 | 11.11 |
| Intraoperative Medications and Fluids | | | | |  |  |
| Dobutamine dose | num | dynamic | μg/kg/min | 0-50 | 99.72 | 99.67 |
| Dopamine dose | num | dynamic | μg/kg/min | 0-50 | 99.91 | 99.89 |
| Ephedrine dose | num | dynamic | mg | 0-100 | 97.66 | 98.15 |
| Epinephrine bolus dose | num | dynamic | μg | 0-10000 | 99.84 | 99.99 |
| Epinephrine infusion dose | num | dynamic | μg/min | 0-100 | 99.84 | 99.93 |
| Milrinone dose | num | dynamic | mg | 0-20 | 99.99 | 99.94 |
| Norepinephrine dose | num | dynamic | μg/kg/min | 0-10 | 99.65 | 99.53 |
| Nitroglycerin dose | num | dynamic | mg | 0-100 | 99.89 | 99.84 |
| Phenylephrine dose | num | dynamic | μg | 0-10000 | 99.85 | 98.62 |
| Vasopressin dose | num | dynamic | U | 0-100 | 99.99 | 99.93 |
| 20% albumin volume | num | dynamic | mL | 0-1000 | 99.86 | 99.83 |
| 5% albumin volume | num | dynamic | mL | 0-2000 | 99.99 | 99.99 |
| Cryoprecipitate units | num | dynamic | unit | 0-100 | 99.96 | 99.95 |
| Fresh frozen plasma units | num | dynamic | unit | 0-50 | 99.86 | 99.62 |
| Platelet concentrate units | num | dynamic | unit | 0-20 | 99.97 | 99.74 |
| Plasma pheresis units | num | dynamic | unit | 0-20 | 99.96 | 99.96 |
| Packed red blood cell units | num | dynamic | unit | 0-50 | 99.46 | 98.71 |
| Blood loss volume | num | dynamic | mL | 0-30000 | 98.79 | 93.84 |
| Urine output | num | dynamic | mL | 0-10000 | 98.99 | 94.92 |
| 10% dextrose in water volume | num | dynamic | mL | 0-3000 | 99.97 | 99.97 |
| 50% dextrose in water volume | num | dynamic | mL | 0-1000 | 99.99 | 99.99 |
| 5% dextrose in water volume | num | dynamic | mL | 0-5000 | 99.98 | 99.98 |
| Hartmann’s solution volume | num | dynamic | mL | 0-5000 | 99.95 | 99.95 |
| Hypertonic saline volume | num | dynamic | mL | 0-5000 | 94.93 | 95.07 |
| Normal saline volume | num | dynamic | mL | 0-20000 | 98.74 | 97.67 |
| Hydroxyethyl starch volume | num | dynamic | mL | 0-5000 | 98.51 | 98.02 |
| Propofol dose | num | dynamic | mg | 0-5000 | 99.81 | 99.97 |
| Midazolam dose | num | dynamic | mg | 0-100 | 99.97 | 99.96 |
| Fentanyl dose | num | dynamic | μg | 0-5000 | 99.77 | 99.74 |
| Sufentanil dose | num | dynamic | μg | 0-1000 | 95.39 | 95.94 |
| Remifentanil dose | num | dynamic | mg | 0-50 | 97.03 | 97.92 |
| Atracurium dose | num | dynamic | mg | 0-500 | 99.95 | 99.95 |

**Table S8**. Proportions of 5-minute resampling intervals with and without a newly recorded source measurement for intraoperative dynamic variables.

|  | HUAXI | | INSPIRE | |
| --- | --- | --- | --- | --- |
|  | Intervals containing at least one source measurement  (%) | Intervals completed during sequence construction  (%) | Intervals containing at least one source measurement  (%) | Intervals completed during sequence construction  (%) |
| Intraoperative Physiologic Monitoring | | | | |
| Air flow | 1.86 | 98.14 | 2.02 | 97.98 |
| End-tidal desflurane concentration | 20.23 | 79.77 | 1 | 99 |
| End-tidal gas concentration | 1.93 | 98.07 | 2.19 | 97.81 |
| End-tidal isoflurane concentration | 0.12 | 99.88 | 0.11 | 99.89 |
| End-tidal sevoflurane concentration | 52.48 | 47.52 | 0.24 | 99.76 |
| Fraction of inspired oxygen | 25.99 | 74.01 | 85.63 | 14.37 |
| Nitrous oxide concentration | 0.06 | 99.94 | 0.06 | 99.94 |
| Oxygen flow | 0.66 | 99.34 | 0.73 | 99.27 |
| Arterial diastolic blood pressure | 60.56 | 39.44 | 63.01 | 36.99 |
| Arterial mean blood pressure | 59.51 | 40.49 | 60.89 | 39.11 |
| Arterial systolic blood pressure | 59.76 | 40.24 | 60.9 | 39.1 |
| Noninvasive diastolic blood pressure | 96.84 | 3.16 | 95.67 | 4.33 |
| Noninvasive mean blood pressure | 96 | 4 | 92.3 | 7.7 |
| Noninvasive systolic blood pressure | 96.92 | 3.08 | 93.05 | 6.95 |
| Bispectral index | 24.45 | 75.55 | 19.32 | 80.68 |
| Body temperature | 5.43 | 94.57 | 89.98 | 10.02 |
| Cerebral regional oxygen saturation | 0.08 | 99.92 | 0.08 | 99.92 |
| Cardiac index | 2.12 | 97.88 | 2.44 | 97.56 |
| Central venous pressure | 3.1 | 96.9 | 28.3 | 71.7 |
| Heart rate | 97.06 | 2.94 | 97.62 | 2.38 |
| Minute ventilation | 35.89 | 64.11 | 89.27 | 10.73 |
| Pulmonary artery diastolic pressure | 4.48 | 95.52 | 4.82 | 95.18 |
| Pulmonary artery mean pressure | 4.71 | 95.29 | 5.18 | 94.82 |
| Pulmonary artery systolic pressure | 4.05 | 95.95 | 4.31 | 95.69 |
| Peripheral oxygen saturation | 98.1 | 1.9 | 97.74 | 2.26 |
| ST segment in lead I | 0.25 | 99.75 | 6.16 | 93.84 |
| ST segment in lead II | 1.97 | 98.03 | 17.52 | 82.48 |
| ST segment in lead III | 3.75 | 96.25 | 4.21 | 95.79 |
| ST segment in lead V5 | 3.02 | 96.98 | 3.51 | 96.49 |
| Stroke volume index | 0.42 | 99.58 | 0.43 | 99.57 |
| End-tidal carbon dioxide | 94.09 | 5.91 | 91.04 | 8.96 |
| Positive end-expiratory pressure | 31.74 | 68.26 | 65.03 | 34.97 |
| Peak inspiratory pressure | 35.56 | 64.44 | 90.19 | 9.81 |
| Mean airway pressure | 35.31 | 64.69 | 59.69 | 40.31 |
| Plateau pressure | 32.05 | 67.95 | 48.16 | 51.84 |
| Respiratory rate | 95.62 | 4.38 | 93.11 | 6.89 |
| Tidal volume | 35.55 | 64.45 | 88.89 | 11.11 |
| Intraoperative Medications and Fluids | | | | |
| Dobutamine dose | 0.28 | 99.72 | 0.33 | 99.67 |
| Dopamine dose | 0.09 | 99.91 | 0.11 | 99.89 |
| Ephedrine dose | 2.34 | 97.66 | 1.85 | 98.15 |
| Epinephrine bolus dose | 0.16 | 99.84 | 0.01 | 99.99 |
| Epinephrine infusion dose | 0.16 | 99.84 | 0.07 | 99.93 |
| Milrinone dose | 0.01 | 99.99 | 0.06 | 99.94 |
| Norepinephrine dose | 0.35 | 99.65 | 0.47 | 99.53 |
| Nitroglycerin dose | 0.11 | 99.89 | 0.16 | 99.84 |
| Phenylephrine dose | 0.15 | 99.85 | 1.38 | 98.62 |
| Vasopressin dose | 0.01 | 99.99 | 0.07 | 99.93 |
| 20% albumin volume | 0.14 | 99.86 | 0.17 | 99.83 |
| 5% albumin volume | 0.01 | 99.99 | 0.01 | 99.99 |
| Cryoprecipitate units | 0.04 | 99.96 | 0.05 | 99.95 |
| Fresh frozen plasma units | 0.14 | 99.86 | 0.38 | 99.62 |
| Platelet concentrate units | 0.03 | 99.97 | 0.26 | 99.74 |
| Plasma pheresis units | 0.04 | 99.96 | 0.04 | 99.96 |
| Packed red blood cell units | 0.54 | 99.46 | 1.29 | 98.71 |
| Blood loss volume | 1.21 | 98.79 | 6.16 | 93.84 |
| Urine output | 1.01 | 98.99 | 5.08 | 94.92 |
| 10% dextrose in water volume | 0.03 | 99.97 | 0.03 | 99.97 |
| 50% dextrose in water volume | 0.01 | 99.99 | 0.01 | 99.99 |
| 5% dextrose in water volume | 0.02 | 99.98 | 0.02 | 99.98 |
| Hartmann’s solution volume | 0.05 | 99.95 | 0.05 | 99.95 |
| Hypertonic saline volume | 5.07 | 94.93 | 4.93 | 95.07 |
| Normal saline volume | 1.26 | 98.74 | 2.33 | 97.67 |
| Hydroxyethyl starch volume | 1.49 | 98.51 | 1.98 | 98.02 |
| Propofol dose | 0.19 | 99.81 | 0.03 | 99.97 |
| Midazolam dose | 0.03 | 99.97 | 0.04 | 99.96 |
| Fentanyl dose | 0.23 | 99.77 | 0.26 | 99.74 |
| Sufentanil dose | 4.61 | 95.39 | 4.06 | 95.94 |
| Remifentanil dose | 2.97 | 97.03 | 2.08 | 97.92 |
| Atracurium dose | 0.05 | 99.95 | 0.05 | 99.95 |

An observed interval was defined as a 5-minute interval containing at least one original source measurement for the corresponding variable. A completed interval was defined as an interval without a newly recorded source measurement that was processed according to the variable-type-specific EMR-LIP procedure. These percentages describe interval-level recording density after construction of the regular time grid and should not be interpreted as patient-level missingness rates or as the proportion of entire trajectories generated synthetically. Continuous internal gaps were handled using linear interpolation, forward filling was used only when a preceding observation was available, categorical missingness was represented using an explicit missing category, and variables without valid intraoperative observations were imputed using training-set statistics.

**Table S9**. Threshold-oriented performance of all models on the HUAXI internal test sets

| Model | Operating point | Threshold | Sensitivity, % (95% CI) | Specificity, % (95% CI) | PPV, % (95% CI) | FP per 1000 (95% CI) | FN, n | FN per 1000 | Alerts per 1000 |
| --- | --- | --- | --- | --- | --- | --- | --- | --- | --- |
| ASA | F1-optimal | -2.5083 | 28.3 (16.4, 40.5) | 99.0 (98.9, 99.2) | 9.4 (5.2, 14.1) | 9.8 (8.2, 11.4) | 38 | 2.58 | 10.8 |
| ASA | Target sensitivity 80% | -4.9937 | 88.7 (78.9, 96.3) | 65.4 (64.5, 66.1) | 0.9 (0.7, 1.2) | 345.2 (337.5, 353.4) | 6 | 0.41 | 348.4 |
| ASA | Target sensitivity 90% | -7.4791 | 100.0 (100.0, 100.0) | 0.4 (0.3, 0.5) | 0.4 (0.3, 0.5) | 992.5 (991.1, 993.8) | 0 | 0.00 | 996.1 |
| LR | Default 0.5 | 0.5000 | 56.6 (43.3, 70.6) | 84.6 (84.0, 85.2) | 1.3 (0.9, 1.8) | 153.6 (147.6, 159.5) | 23 | 1.56 | 155.7 |
| LR | F1-optimal | 0.9653 | 28.3 (16.3, 40.7) | 98.2 (98.0, 98.4) | 5.3 (2.8, 8.1) | 18.2 (16.1, 20.4) | 38 | 2.58 | 19.3 |
| LR | Target sensitivity 80% | 0.0886 | 81.1 (70.0, 91.2) | 77.5 (76.9, 78.2) | 1.3 (0.9, 1.7) | 223.9 (217.0, 230.5) | 10 | 0.68 | 226.9 |
| LR | Target sensitivity 90% | 0.0200 | 90.6 (82.0, 97.8) | 68.7 (67.9, 69.4) | 1.0 (0.8, 1.3) | 312.2 (304.6, 319.5) | 5 | 0.34 | 315.4 |
| XGBoost | Default 0.5 | 0.5000 | 56.6 (43.3, 70.6) | 86.4 (85.8, 86.9) | 1.5 (1.0, 2.0) | 135.9 (130.3, 141.7) | 23 | 1.56 | 138.0 |
| XGBoost | F1-optimal | 0.9653 | 28.3 (16.3, 40.7) | 98.4 (98.2, 98.6) | 6.1 (3.2, 9.4) | 15.5 (13.6, 17.7) | 38 | 2.58 | 16.5 |
| XGBoost | Target sensitivity 80% | 0.0886 | 81.1 (70.0, 91.2) | 79.2 (78.5, 79.8) | 1.4 (1.0, 1.8) | 207.5 (201.0, 214.3) | 10 | 0.68 | 210.4 |
| XGBoost | Target sensitivity 90% | 0.0200 | 90.6 (82.0, 97.8) | 70.1 (69.4, 70.9) | 1.1 (0.8, 1.4) | 297.7 (290.4, 305.2) | 5 | 0.34 | 301.0 |
| LSTM | Default 0.5 | 0.5000 | 56.6 (43.3, 70.6) | 93.2 (92.8, 93.6) | 2.9 (1.9, 4.0) | 67.5 (63.6, 71.8) | 23 | 1.56 | 69.5 |
| LSTM | F1-optimal | 0.9653 | 26.4 (14.5, 38.9) | 98.9 (98.8, 99.1) | 8.2 (4.1, 12.6) | 10.6 (8.9, 12.3) | 39 | 2.64 | 11.5 |
| LSTM | Target sensitivity 80% | 0.0891 | 81.1 (70.2, 91.4) | 79.0 (78.3, 79.7) | 1.4 (1.0, 1.8) | 209.1 (202.4, 215.7) | 10 | 0.68 | 212.0 |
| LSTM | Target sensitivity 90% | 0.0226 | 90.6 (82.0, 97.8) | 69.1 (68.3, 69.8) | 1.0 (0.8, 1.3) | 308.4 (300.6, 316.0) | 5 | 0.34 | 311.6 |
| PRIME-MoE | Default 0.5 | 0.5000 | 62.3 (48.9, 75.9) | 95.5 (95.1, 95.8) | 4.7 (3.2, 6.5) | 45.2 (41.8, 48.5) | 20 | 1.36 | 47.5 |
| PRIME-MoE | F1-optimal | 0.9860 | 18.9 (9.3, 29.8) | 99.6 (99.5, 99.7) | 14.7 (6.7, 23.7) | 3.9 (2.9, 5.0) | 43 | 2.92 | 4.6 |
| PRIME-MoE | Target sensitivity 80% | 0.0250 | 81.1 (70.0, 91.4) | 87.2 (86.7, 87.8) | 2.2 (1.6, 2.9) | 127.2 (122.0, 132.3) | 10 | 0.68 | 130.1 |
| PRIME-MoE | Target sensitivity 90% | 0.0036 | 90.6 (81.6, 98.0) | 76.4 (75.7, 77.1) | 1.4 (1.0, 1.8) | 235.1 (228.1, 242.2) | 5 | 0.34 | 238.4 |

Positive class = postoperative in-hospital death. A procedure was classified as positive when the model risk score was greater than or equal to the specified threshold.

Abbreviations: ASA, American Society of Anesthesiologists physical status score; CI, confidence interval; FN, false negative; FP, false positive; LR, logistic regression; LSTM, long short-term memory; PPV, positive predictive value; PRIME-MoE, Postoperative Risk Modeling Expert network. Point estimates and 95% confidence intervals were obtained from 2000 bootstrap resamples where shown. Target-sensitivity operating points represent the thresholds yielding approximately 80% or 90% sensitivity. Alert burden was defined as the total number of predicted-positive procedures per 1000 procedures.

**Table S10**. Threshold-oriented performance of all models on the INSPIRE internal test sets

| Model | Operating point | Threshold | Sensitivity, % (95% CI) | Specificity, % (95% CI) | PPV, % (95% CI) | FP per 1000 (95% CI) | FN, n | FN per 1000 | Alerts per 1000 |
| --- | --- | --- | --- | --- | --- | --- | --- | --- | --- |
| ASA | F1-optimal | -0.0297 | 20.0 (13.1, 28.0) | 100.0 (99.9, 100.0) | 73.3 (56.7, 88.9) | 0.4 (0.2, 0.7) | 88 | 4.59 | 1.6 |
| ASA | Target sensitivity 80% | -5.7429 | 94.5 (90.1, 98.2) | 36.0 (35.4, 36.7) | 0.8 (0.7, 1.0) | 635.8 (629.0, 642.5) | 6 | 0.31 | 641.3 |
| ASA | Target sensitivity 90% | -5.7429 | 94.5 (90.1, 98.2) | 36.0 (35.4, 36.7) | 0.8 (0.7, 1.0) | 635.8 (629.0, 642.5) | 6 | 0.31 | 641.3 |
| LR | Default 0.5 | 0.5000 | 60.9 (52.9, 69.4) | 77.0 (76.4, 77.6) | 1.7 (1.4, 2.1) | 228.5 (222.6, 234.3) | 50 | 2.53 | 232.4 |
| LR | F1-optimal | 0.9997 | 31.2 (23.5, 39.7) | 99.6 (99.5, 99.7) | 32.0 (24.4, 40.7) | 4.3 (3.4, 5.2) | 88 | 4.46 | 6.3 |
| LR | Target sensitivity 80% | 0.0574 | 80.5 (73.5, 87.1) | 74.7 (74.1, 75.3) | 2.0 (1.7, 2.4) | 251.7 (245.6, 257.8) | 25 | 1.27 | 256.9 |
| LR | Target sensitivity 90% | 0.0014 | 90.6 (85.5, 95.3) | 65.6 (64.9, 66.3) | 1.7 (1.4, 2.0) | 341.5 (335.1, 348.3) | 12 | 0.61 | 347.4 |
| XGBoost | Default 0.5 | 0.5000 | 62.5 (54.3, 70.8) | 81.7 (81.1, 82.2) | 2.2 (1.7, 2.7) | 182.0 (176.7, 187.5) | 48 | 2.43 | 186.0 |
| XGBoost | F1-optimal | 0.9997 | 29.7 (22.5, 38.2) | 99.6 (99.5, 99.7) | 31.4 (23.6, 40.0) | 4.2 (3.3, 5.1) | 90 | 4.56 | 6.1 |
| XGBoost | Target sensitivity 80% | 0.0814 | 80.5 (73.5, 87.1) | 79.6 (79.0, 80.2) | 2.5 (2.1, 3.0) | 202.5 (197.0, 208.3) | 25 | 1.27 | 207.7 |
| XGBoost | Target sensitivity 90% | 0.0014 | 90.6 (85.5, 95.3) | 69.6 (68.9, 70.2) | 1.9 (1.6, 2.3) | 302.3 (296.2, 308.7) | 12 | 0.61 | 308.2 |
| LSTM | Default 0.5 | 0.5000 | 66.4 (58.3, 74.8) | 86.5 (86.1, 87.0) | 3.1 (2.5, 3.8) | 133.7 (128.9, 138.6) | 43 | 2.18 | 138.0 |
| LSTM | F1-optimal | 0.9998 | 28.9 (21.2, 37.4) | 99.6 (99.6, 99.7) | 34.6 (26.1, 44.1) | 3.5 (2.7, 4.4) | 91 | 4.61 | 5.4 |
| LSTM | Target sensitivity 80% | 0.1024 | 80.5 (73.5, 87.1) | 84.8 (84.2, 85.2) | 3.3 (2.7, 4.0) | 151.5 (146.5, 156.6) | 25 | 1.27 | 156.7 |
| LSTM | Target sensitivity 90% | 0.0014 | 90.6 (85.5, 95.3) | 73.8 (73.1, 74.4) | 2.2 (1.8, 2.6) | 260.5 (254.5, 266.7) | 12 | 0.61 | 266.4 |
| PRIME-MoE | Default 0.5 | 0.5000 | 73.4 (65.9, 81.1) | 94.8 (94.5, 95.1) | 8.5 (6.9, 10.2) | 51.5 (48.4, 54.8) | 34 | 1.72 | 56.3 |
| PRIME-MoE | F1-optimal | 0.9982 | 44.5 (35.8, 53.3) | 99.2 (99.1, 99.3) | 26.1 (20.4, 32.1) | 8.2 (6.9, 9.4) | 71 | 3.60 | 11.0 |
| PRIME-MoE | Target sensitivity 80% | 0.3395 | 80.5 (73.8, 87.2) | 94.0 (93.7, 94.4) | 8.1 (6.7, 9.6) | 59.4 (56.1, 62.8) | 25 | 1.27 | 64.6 |
| PRIME-MoE | Target sensitivity 90% | 0.0028 | 90.6 (85.3, 95.4) | 80.5 (80.0, 81.1) | 2.9 (2.5, 3.5) | 193.6 (188.2, 199.2) | 12 | 0.61 | 199.5 |

Positive class = postoperative in-hospital death. A procedure was classified as positive when the model risk score was greater than or equal to the specified threshold.

Abbreviations: ASA, American Society of Anesthesiologists physical status score; CI, confidence interval; FN, false negative; FP, false positive; LR, logistic regression; LSTM, long short-term memory; PPV, positive predictive value; PRIME-MoE, Postoperative Risk Modeling Expert network. Point estimates and 95% confidence intervals were obtained from 2000 bootstrap resamples where shown. Target-sensitivity operating points represent the thresholds yielding approximately 80% or 90% sensitivity. Alert burden was defined as the total number of predicted-positive procedures per 1000 procedures.

**Additional Supplemental Figures**

**Figure S2**. Reliability diagrams of PRIME-MoE in internal and reciprocal cross-database evaluations. (A) INSPIRE internal test set; (B) HUAXI internal test set; (C) INSPIRE-trained model evaluated in HUAXI; (D) HUAXI-trained model evaluated in INSPIRE. The dashed diagonal line represents perfect calibration, and the observed curve shows the observed event rate across predicted-probability bins.

**Figure S3**. Mean PRIME-MoE router weights across ASA physical status classes in the INSPIRE and HUAXI databases. (A) INSPIRE database; (B) HUAXI database. Each cell represents the mean routing weight assigned to the corresponding expert within each ASA physical status class.

**Additional Supplemental Tables**

**Table S11**. Calibration metrics for PRIME-MoE

| Setting | N (events) | Prevalence | AUROC | AUPRC | Brier (95% CI) | Slope | Intercept | ECE |
| --- | --- | --- | --- | --- | --- | --- | --- | --- |
| INSPIRE internal | 19743 (128) | 0.65% | 0.937 | 0.222 | 0.045 (0.042-0.048) | 0.35 | -4.06 | 0.054 |
| HUAXI internal | 14749 (53) | 0.36% | 0.907 | 0.064 | 0.035 (0.033-0.038) | 0.40 | -4.11 | 0.048 |
| INSPIRE to HUAXI | 14749 (53) | 0.36% | 0.878 | 0.081 | 0.048 (0.046-0.052) | 0.28 | -4.63 | 0.063 |
| HUAXI to INSPIRE | 19743 (128) | 0.65% | 0.908 | 0.128 | 0.044 (0.041-0.047) | 0.49 | -3.85 | 0.067 |

Note: Ideal calibration slope is 1 and intercept is 0. ECE was calculated using 10 equal-width bins.

Table S12A. Trainable parameter counts

| Model | Trainable parameters |
| --- | --- |
| PRIME-MoE (K=1, LSTM expert h=128) | 196,066 |
| PRIME-MoE (K=2, LSTM expert h=128) | 352,387 |
| PRIME-MoE (K=3, LSTM expert h=128) | 508,708 |
| PRIME-MoE (K=4, LSTM expert h=128) | 665,029 |
| PRIME-MoE (K=5, LSTM expert h=128) | 821,350 |
| LSTM baseline (mlp+lstm h=128) | 238,079 |
| LSTM baseline (mlp+lstm h=256) | 555,775 |

Table S12B. Synthetic GPU inference latency at batch size 1

| Model | Seq 20, ms | Seq 40, ms | Seq 80, ms |
| --- | --- | --- | --- |
| PRIME-MoE K=4 | 3.8 | 7.2 | 13.2 |
| PRIME-MoE K=1 | 1.2 | 2.0 | 3.4 |
| LSTM baseline h=256 | 1.0 | 1.8 | 3.3 |
| LSTM baseline h=128 | 1.0 | 2.2 | 3.3 |

Table S12C. Real-data latency for PRIME-MoE K=4 on 300 INSPIRE test cases

| Metric | Value |
| --- | --- |
| Mean | 13.1 ms/case |
| Median | 11.4 ms/case |
| P95 | 25.8 ms/case |
| P99 | 33.8 ms/case |

Note: Device was an NVIDIA L20 GPU. Timings exclude electronic health record extraction and EMR-LIP preprocessing.

Table S13A. Mean router weights by surgical site in INSPIRE

| Surgery site | N (events) | Expert 0 | Expert 1 | Expert 2 | Expert 3 |
| --- | --- | --- | --- | --- | --- |
| Abdomen | 4370 (18) | 0.742 | 0.086 | 0.107 | 0.066 |
| CNS | 913 (15) | 0.681 | 0.089 | 0.136 | 0.093 |
| Cardiovascular | 528 (6) | 0.597 | 0.156 | 0.119 | 0.128 |
| Head and Neck | 3204 (10) | 0.802 | 0.063 | 0.073 | 0.062 |
| Musculoskeletal | 1914 (15) | 0.662 | 0.095 | 0.130 | 0.112 |
| Other | 2301 (19) | 0.758 | 0.070 | 0.100 | 0.073 |
| Respiratory | 883 (13) | 0.626 | 0.105 | 0.131 | 0.139 |
| Skin and Soft Tissue | 2611 (12) | 0.811 | 0.059 | 0.068 | 0.061 |
| Urinary and Reproductive | 3019 (20) | 0.815 | 0.064 | 0.061 | 0.060 |

Note: Chi-square test of surgical site by dominant expert: chi-square=822.7, df=24, P<.001.

Table S13B. Mean router weights by surgical site in HUAXI

| Surgery site | N (events) | Expert 0 | Expert 1 | Expert 2 | Expert 3 |
| --- | --- | --- | --- | --- | --- |
| Abdomen | 7503 (27) | 0.452 | 0.277 | 0.099 | 0.172 |
| CNS | 134 (2) | 0.423 | 0.259 | 0.130 | 0.188 |
| Cardiovascular | 416 (9) | 0.280 | 0.273 | 0.227 | 0.220 |
| Head and Neck | 647 (4) | 0.401 | 0.301 | 0.073 | 0.225 |
| Musculoskeletal | 2295 (3) | 0.379 | 0.329 | 0.116 | 0.175 |
| Other | 179 (5) | 0.347 | 0.302 | 0.122 | 0.229 |
| Respiratory | 1833 (3) | 0.473 | 0.281 | 0.087 | 0.158 |
| Skin and Soft Tissue | 487 (0) | 0.444 | 0.267 | 0.087 | 0.203 |
| Urinary and Reproductive | 1255 (0) | 0.463 | 0.278 | 0.078 | 0.180 |

Note: Chi-square test of surgical site by dominant expert: chi-square=1272.4, df=24, P<.001.

Table S14. End-of-surgery and 30-minute-before-end performance

| Database | Horizon | AUROC | AUPRC | Brier | Calibration slope |
| --- | --- | --- | --- | --- | --- |
| INSPIRE | End of surgery | 0.937 | 0.222 | 0.045 | 0.35 |
| INSPIRE | 30 min before end | 0.931 | 0.205 | 0.048 | 0.36 |
| HUAXI | End of surgery | 0.907 | 0.064 | 0.035 | 0.40 |
| HUAXI | 30 min before end | 0.902 | 0.065 | 0.038 | 0.41 |

Note: The final six 5-minute bins were removed without retraining. Short operations retained at least one time bin.

Table S15A. PRIME-MoE operating points in INSPIRE

| Rule | Threshold | Sensitivity | Specificity | PPV | NPV | Alert rate | FP/1000 | F1 |
| --- | --- | --- | --- | --- | --- | --- | --- | --- |
| Youden | 0.3239 | 81.2% | 94.0% | 8.1% | 99.9% | 6.5% | 60 | 0.147 |
| F1 optimal | 0.9982 | 44.5% | 99.2% | 26.1% | 99.6% | 1.1% | 8 | 0.329 |
| Sens>=0.80 | 0.3395 | 80.5% | 94.0% | 8.1% | 99.9% | 6.5% | 60 | 0.147 |
| Sens>=0.90 | 0.00285 | 90.6% | 80.5% | 2.9% | 99.9% | 19.9% | 195 | 0.057 |
| Sens>=0.95 | 0.0007411 | 95.3% | 71.6% | 2.1% | 100.0% | 28.9% | 284 | 0.042 |
| fixed 0.01 | 0.01 | 85.2% | 85.8% | 3.8% | 99.9% | 14.6% | 142 | 0.072 |
| fixed 0.05 | 0.05 | 84.4% | 90.1% | 5.3% | 99.9% | 10.4% | 99 | 0.099 |
| fixed 0.1 | 0.1 | 82.8% | 91.6% | 6.0% | 99.9% | 8.9% | 84 | 0.112 |
| fixed 0.2 | 0.2 | 81.2% | 93.0% | 7.0% | 99.9% | 7.5% | 70 | 0.130 |
| fixed 0.5 | 0.5 | 73.4% | 94.8% | 8.5% | 99.8% | 5.6% | 52 | 0.152 |

Table S15B. PRIME-MoE operating points in HUAXI

| Rule | Threshold | Sensitivity | Specificity | PPV | NPV | Alert rate | FP/1000 | F1 |
| --- | --- | --- | --- | --- | --- | --- | --- | --- |
| Youden | 0.01766 | 86.8% | 85.7% | 2.1% | 99.9% | 14.5% | 143 | 0.042 |
| F1 optimal | 0.986 | 18.9% | 99.6% | 14.7% | 99.7% | 0.5% | 4 | 0.165 |
| Sens>=0.80 | 0.02497 | 81.1% | 87.2% | 2.2% | 99.9% | 13.0% | 128 | 0.044 |
| Sens>=0.90 | 0.003644 | 90.6% | 76.4% | 1.4% | 100.0% | 23.8% | 236 | 0.027 |
| Sens>=0.95 | 0.000314 | 96.2% | 43.9% | 0.6% | 100.0% | 56.3% | 561 | 0.012 |
| fixed 0.01 | 0.01 | 86.8% | 82.8% | 1.8% | 99.9% | 17.5% | 172 | 0.035 |
| fixed 0.05 | 0.05 | 69.8% | 89.6% | 2.4% | 99.9% | 10.6% | 104 | 0.046 |
| fixed 0.1 | 0.1 | 69.8% | 91.7% | 3.0% | 99.9% | 8.5% | 83 | 0.057 |
| fixed 0.2 | 0.2 | 66.0% | 93.3% | 3.5% | 99.9% | 6.9% | 67 | 0.066 |
| fixed 0.5 | 0.5 | 62.3% | 95.5% | 4.7% | 99.9% | 4.7% | 45 | 0.088 |

Note: F1-maximizing thresholds are reported for completeness and are not proposed as universal clinical cutoffs. Thresholds should be selected and frozen on a local validation set after recalibration.
